# Shifting reef fish assemblages along a depth gradient in Pohnpei, Micronesia

**DOI:** 10.7717/peerj.4650

**Published:** 2018-04-24

**Authors:** Richard R. Coleman, Joshua M. Copus, Daniel M. Coffey, Robert K. Whitton, Brian W. Bowen

**Affiliations:** 1 Hawai’i Institute of Marine Biology, Kāne’ohe, Hawai’i, United States of America; 2 Department of Biology, University of Hawai’i at Mānoa, Honolulu, Hawai’i, United States of America; 3 Bernice P. Bishop Museum, Honolulu, Hawai’i, United States of America

**Keywords:** Deep reef refugia hypothesis, Depth distribution, Mesophotic coral ecosystem, Trophic, Fish surveys, Reef fishes

## Abstract

Mesophotic coral ecosystems (MCEs) continue to be understudied, especially in island locations spread across the Indo-Pacific Ocean. Pohnpei is the largest island in the Federated States of Micronesia, with a well-developed barrier reef, and steep slopes that descend to more than 1,000 m. Here we conducted visual surveys along a depth gradient of 0 to 60 m in addition to video surveys that extend to 130 m, with 72 belt transects and 12 roving surveys using closed-circuit rebreathers, to test for changes in reef fish composition from shallow to mesophotic depths. We observed 304 fish species across 47 families with the majority confined to shallow habitat. Taxonomic and trophic positions at 30 m showed similar compositions when compared against all other depths. However, assemblages were comprised of a distinct shallow (<30 m) and deep (>30 m) group, suggesting 30 m as a transition zone between these communities. Shallow specialists had a high probability of being herbivores and deep specialists had a higher probability of being planktivores. Acanthuridae (surgeonfishes), Holocentridae (soldierfishes), and Labridae (wrasses) were associated primarily with shallow habitat, while Pomacentridae (damselfishes) and Serranidae (groupers) were associated with deep habitat. Four species may indicate Central Pacific mesophotic habitat: *Chromis circumaurea, Luzonichthys seaver, Odontanthias borbonius,* and an undescribed slopefish (*Symphysanodon sp.*). This study supports the 30 m depth profile as a transition zone between shallow and mesophotic ecosystems (consistent with accepted definitions of MCEs), with evidence of multiple transition zones below 30 m. Disturbances restricted to either region are not likely to immediately impact the other and both ecosystems should be considered separately in management of reefs near human population centers.

## Introduction

Studies of coral reef ecosystems are almost exclusively limited to depths shallower than 30 m, well above the lower depth limit of most photosynthetic coral (Kahng, Copus & Wagner, 2014; Menza, Kendall & Hile, 2008). This bias towards the upper reaches of coral reef ecosystems reflects the technological limitations and physiological constraints on SCUBA divers that restricts them to the upper 50 m of the water column (Pyle, 1998). Below this zone lie the largely unexplored mesophotic coral ecosystems (MCEs), the light-dependent communities that typically range from depths of 30 m to over 150 m in tropical and subtropical regions (Kahng, Copus & Wagner, 2014). The dominant biotic cover in the mesophotic zone is coral, sponge, or algae (Puglise et al., 2009). Although there has been a surge in scientific interest about MCEs, it remains logistically challenging to conduct field research at such depths, especially in remote locations. As such, information on the ecology and community composition of MCEs remains scarce (Kahng et al., 2010), based largely on studies from a few key locations with adequate technical support (Kahng, Copus & Wagner, 2014; Pyle et al., 2016; Wagner et al., 2014).

Mesophotic reef fish surveys to date have been conducted almost entirely with remotely operated vehicles (ROVs), cameras placed on the reef, and submersibles (Dennis & Bright, 1988; Lukens, 1981; Malcolm, Jordan & Smith, 2011; Sackett et al., 2014; Thresher & Colin, 1986). These technologies have produced many valuable scientific advances but there are limitations including the overall expense (especially for submersibles and boat time), limited taxonomic scope, avoidance or attraction bias, and the logistic challenges of making comparable measurements across study sites (Clarke et al., 2010). Because of these technological limitations, our understanding of reef biota at depths of 50–150 m remains grievously inadequate (Feitoza, Rosa & Rocha, 2005; Kahng, Copus & Wagner, 2014; Kahng et al., 2010; Pyle, 1996; Pyle, 2000). To date, these efforts have been applied to MCEs in a few regions including Hawai‘i, Australia, and the Caribbean (Bridge et al., 2011; Kahng, Copus & Wagner, 2016; Kahng & Kelley, 2007; Menza, Kendall & Hile, 2008; Rooney et al., 2010).

With the development of closed circuit rebreather (CCR) technology, diver surveys of mesophotic reef fishes became feasible (at least to depths of 160–180 m, the lower depth at which CCR technologies can effectively be utilized); however, the geographic distribution of CCR surveys to date is limited to a few locations: Hawai’i, the Caribbean, the South Atlantic Ocean, the Red Sea, and the Gulf of Mexico (e.g., Andradi-Brown et al., 2016; De Oliveira Soares et al., 2016; Fukunaga et al., 2016; Kosaki et al., 2016; Pinheiro et al., 2016; Simon et al., 2016; Harter et al., 2017). Systematic surveys of fish fauna by depth are very limited (but see Pinheiro et al., 2016). As a result, the depth distributions of reef fishes are largely based on data using SCUBA at shallower depths <40 m.

Given the dearth of MCE surveys, it is likely that many of the currently recognized depth ranges of coral reef organisms are an artifact of diver physiology and technology rather than habitat preference. Therefore, depth distributions of many marine species, especially in remote locations, will continue to be uncertain until MCEs have been thoroughly surveyed (Kahng, Copus & Wagner, 2014). Moreover, open-circuit SCUBA has inherent biases due to the noise and visual impairment of emitting bubbles into the environment that may lead to an underestimate of species richness and abundance compared to CCR surveys (Gray et al., 2016; Lindfield et al., 2014).

Understanding the mechanisms that regulate the depth distribution of marine species is essential to the analysis of community-level responses to anthropogenic stressors. The deep reef refugia hypothesis (DRRH) states that deep reefs are less impacted by anthropogenic stressors that plague shallow water reefs, and can act as a source of reproductive input to repopulate shallow reefs following a disturbance (Bongaerts et al., 2010). A key element to the DRRH is that shallow species inhabit the deep reefs, below the impacted zone. Therefore, an understanding of the lower depth limits of marine species is crucial to assessing the resilience of these communities.

Previous studies indicate a trophic shift with depth on MCEs in many locations (Kahng, Copus & Wagner, 2016 and references therein). For example, isotopic analyses of fish species occupying both shallow and deep reefs in Hawai’i show a shift with increasing depth to a higher trophic position for benthic omnivores and invertivores, but not for planktivores (Bradley et al., 2016). Growth and body condition of individuals occupying mesophotic vs. shallow habitat has also been shown to vary by geographic location rather than by depth (Winston, Taylor & Franklin, 2017).

Here we describe surveys of fish community assemblages in a Micronesian coral reef ecosystem at depths ranging from 0 to 130 m, to elucidate how coral reef fish communities utilize shallow and mesophotic zones. Pohnpei hosts a well-developed barrier reef and inner lagoon, and may be a stepping stone for marine species to colonize the Pacific (George et al., 2008). The rich habitats of Pohnpei support over 650 fish species and approximately 330 coral species (Allen, Steene & Allen, 2005; Turak & DeVantier, 2005). Pohnpei is characterized by steep slopes and ledges that descend to depths of more than 1,000 m (Krüger & Kumar, 2008) providing an ideal location to identify the transition between shallow and deep reef fish communities, and to explore the ecological characteristics influencing such transitions. Documenting such habitat shifts of reef fishes is an essential foundation for understanding mesophotic ecosystems, refuge hypotheses, and management of shallow and mesophotic coral reef ecosystems. This study aims to identify the depth range where the shift between shallow and mesophotic ecosystems occurs in relationship to fish assemblages, as well as identify the ecological drivers that facilitate the shift between these environments.

## Material and Methods

### Sampling design

**Figure 1:**
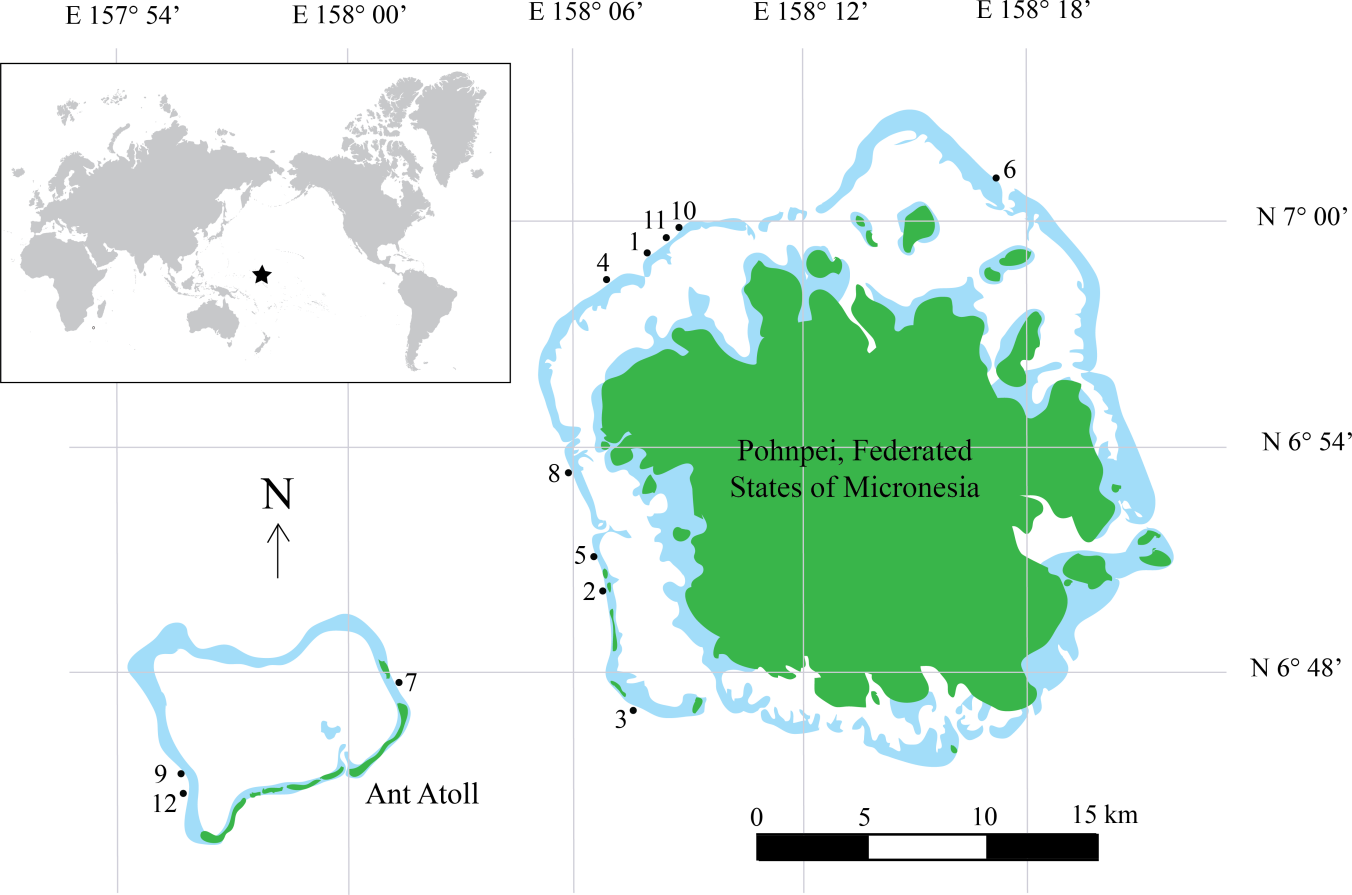
Map of Pohnpei, FSM. Filled dark green colors represent terrestrial habitat while lighter blue areas indicate shallow reefs (depth <5 m). Filled circles represent locations of surveys. Inset shows geographic location of Pohnpei, indicated by the star.

Underwater fish surveys were conducted during July 3–July 15, 2014 using CCR technology. We surveyed conspicuous, diurnal fishes on the outer slope of the island of Pohnpei, Federated States of Micronesia, and adjacent Ant Atoll located less than 15 km to the west. A total of 12 sites were surveyed (Fig. 1, Table S1) by divers using Inspiration CCRs (Ambient Pressure Diving, Ltd., Helston, Cornwall, UK) and breathing tri-mix gas. CCRs are superior to open-circuit SCUBA for this type of study for several reasons: (1) they eliminate expelled gas into the environment and subsequently reduce noise that affects fish behaviors (Lindfield et al., 2014); (2) they greatly reduce gas consumption allowing extended bottom times and lower associated costs compared to open-circuit SCUBA; and (3) they function on a fixed O_2_ partial pressure, reducing decompression obligation and increasing the bottom time and number of transects on a single dive. Research involving vertebrate animals used in this study was in compliance with the Institutional Animal Care and Use Committee (IACUC) policies and approved by the University of Hawaii’s Regulatory Compliance Office under protocol # 09 − 753 − 6.

### Diver transects

Standard commercial depth finders were used to locate slopes, ledges and other fish habitat features to depths >130 m. Visibility of up to 50 m was typical and allowed for surface visual assessment, by snorkel before the dive, to identify similar bathymetric reliefs and to ensure habitat consistency between sites based on the knowledge and previous experience of the divers. Transect surveys were conducted at depths of 10, 20, 30, 40, 50, and 60 m, with a total of 12 transects per depth. Conspicuous, diurnally active reef fishes were counted and recorded using a visual belt transect survey method (Brokovich et al., 2008). Divers swam along a 25 m × 5 m belt transect and identified and counted all fish encountered to the lowest possible taxon. The number of individual schooling fish that entered the 5 m diameter of the survey were estimated and recorded. A second diver video-recorded all transects to aid identification of rare or unusual specimens. Cryptic species, those that were too difficult to identify, were not recorded or included in any analyses. For trophic comparisons, fishes were grouped into six guilds based on Myers (1989), Allen, Steene & Allen (1998), Lieske & Myers (2002), and Randall (2005): planktivores, sessile invertebrate feeders, piscivores, mobile invertebrate feeders, herbivores, and corallivores. When dietary information was not available the trophic assignment from the majority of congeners was used.

We used a permutation multivariate analysis of variance (PERMANOVA; Anderson, 2006) and permutation analysis of dispersion (PERMDISP), as implemented in the statistical software Primer v 6.1.13 with Permanova+ v 1.0.3, to investigate differences in fish community assemblages between depths. PERMANOVA is used to test dissimilarity within a priori groups as compared to between groups and PERMDISP is used to determine whether there are differences in dispersions (variance) among a priori groups. Abundance data was normalized with a square-root transformation. This approach increases the precision with which we measure the differences for rarer species by reducing the contribution of highly abundant species in relation to less abundant species. After data normalization, separate analyses were conducted for two taxonomical groups (by species and family) and one functional grouping (by trophic guild). At the family level, we were interested in ecological comparisons, therefore, subfamilies that are ecologically divergent from the main members of their family (i.e., subfamily Anthiadinae (Serranidae), subfamily Caesioninae (Lutjanidae), subfamily Scarinae (Labridae)) were analyzed separately. For brevity, “family” and “families” are used hereafter to include taxonomic families and these three subfamilies. The PERMANOVA and PERMDSIP analyses, using an unrestricted permutation of raw data (9,999 permutations) and a partial sum of squares (Type III), were based on a zero-adjusted Bray–Curtis resemblance similarity matrix of the species abundance for each variable (Clarke & Gorley, 2006). A dummy variable equal to 1 was added to the matrix to address the problem of highly fluctuating similarity values. Instead of two samples containing values of zero being undefined, the zero-adjusted matrix defines these samples as 100% similar because they share the dummy variable (Clarke & Gorley, 2006). To test for differences in community assemblages for each variable along a depth gradient, a PERMANOVA pairwise comparison test was conducted. False discovery rates were controlled and maintained at a *α* = 0.05 among all pairwise tests to account for multiple comparisons (Benjamini & Yekutieli, 2001; Narum, 2006). A similarity percentage (SIMPER) analysis was conducted to identify the variables that are contributing the most at each depth by calculating the average percent contribution of each variable based on the Bray–Curtis resemblance similarity matrix. Variables that contributed at least 75% of the difference between groups were recorded.

Data were visualized and assessed using a canonical analysis of principle coordinates (CAP) in the program Primer v6 with Permanova+. CAP is useful to discriminate when there are *a priori* known differences between groups (discriminant analysis) and to determine a correlation between variables (canonical correlation) (Anderson & Willis, 2003).

For each depth, a sample-based rarefaction curve and species richness were estimated using EstimateS v.9.1.0 (http://purl.oclc.org/estimates; Colwell, 2013). Michaelis–Menten richness estimator computations were used to create a sample-based rarefaction curve which estimated the total number of surveys that needed to be conducted to identify the entire community (Colwell & Coddington, 1994). Simulations were based on 100 randomizations without replacement and extrapolated to 100 samples for each depth. Two different methods were used to calculate a conservative estimate of species richness for each depth, Chao1 and Abundance Coverage-based Estimator (ACE) (Chao & Lee, 1992; Colwell & Coddington, 1994; Shen, Chao & Lin, 2003). Chao1 is a robust estimator of minimum species richness and ACE incorporates sample coverage to estimate the proportion of assemblage richness represented by species in a single sample (Gotelli & Colwell, 2011).

### Video surveys

Due to logistical constraints of diver-led transects with increasing depth, such as limited bottom time, non-standardized roving diver surveys were simultaneously conducted using a single underwater video camera (Sony Model No. PXW-Z100 XDCAM 4K with Gates Z100 housing; Sony, Tokyo, Japan; Gates, Poway, CA, USA). Video surveys were conducted at the same sites as the transect surveys for a total of 12 surveys. Once the maximum depth was reached, the videographer would begin recording the fish community. All video recordings followed the decompression profile as the diver moved to the surface, allowing for a higher amount of time surveying as depth decreased. As dives could last up to five hours (exceeding video battery life) recordings were taken with an effort to document all species encountered within each depth range. As a result, no attempt was made to standardize recording time by depth but instead fish were recorded opportunistically with decompression ceilings which varied by dive. Total dive time spent between each depth range is summarized in Table S2. As survey depth increased, the abundance of fish decreased, allowing for robust documentation of the representative fish community at these depths.

Using data obtained from video surveys, the fish assemblage was divided into ‘shallow specialists’ and ‘deep specialists’ based on their occurrence on either side of 30, 40, 50, and 60 m depth contours. Shallow specialists occurred exclusively above, and deep specialists below the specified depth contour per Bridge et al. (2016). Species that occurred both above and below the specified depth contour (i.e., ‘depth-generalists’) were removed prior to the analysis. Relatively few species (*n* = 38) occurred exclusively at depths below 70 m; therefore, depth contours used to divide shallow and deep specialists below 60 m were not analyzed to avoid model convergence issues. The effect of trophic guild on depth-specialism was analyzed using generalized linear mixed-effects models (GLMMs) with a binomial distribution (0, shallow specialists; 1, deep specialists) and a logit link function using each specified depth contour. In order to improve model convergence, sessile invertebrate feeders, mobile invertebrate feeders, and corallivores were combined into a single ‘carnivore’ trophic guild. Taxon (genus nested within family) was included as a random variable to account for the non-independence of shared ancestry per Bridge et al. (2016). GLMMs were constructed in R using the ‘lme4’ package (Bates et al., 2015) and compared against the null model using a maximum likelihood ratio test (*χ*^2^, *P* < 0.05).

## Results

### Survey summary and species richness

Video surveys revealed 302 fish species representing 47 families at depths of 0–130 m (Table 1). The most common families were Labridae (wrasses) and Pomacanthidae (damselfishes) while several families were represented by a single species, including Ephippidae (spadefishes), Haemulidae (grunts) and Symphysanodontidae (slopefishes). Diver transect surveys at depths of 10–60 m yielded a much lower number of taxa at 120 fish species representing 27 families (Table S3). The most abundant species were *Chromis ternatensis* (Pomacentridae), *Ctenochaetus striatus* (Acanthuridae), *Pseudanthias pascalus* (Serranidae, subfamily Anthiadinae), and *Chromis alpha*.

**Table 1:** Species checklist by depth (0–130 m). Checklist of fish species surveyed between 0–130 m on the island of Pohnpei, Federated States of Micronesia.

		Depth (m)												
Species	Trophic Guild	0–10	11–20	21–30	31–40	41–50	51–60	61–70	71–80	81–90	91–100	101–110	111–120	121–130
Ginglymostomatidae														
*Nebrius ferrugineus*	MIF	X												
Carcharhinidae														
*Carcharhinus melanopterus*	Ps	X												
*Triaenodon obesus*	Ps	X	X	X	X	X	X							
Dasyatidae														
*Taeniurops meyeni*	MIF										X			
Muraenidae														
*Gymnothorax flavimarginatus*	Ps	X	X											
*Gymnothorax javanicus*	Ps		X											
*Gymnothorax nudivomer*	Ps							X	X					
Synodontidae														
*Synodus variegatus*	Ps	X	X	X										
Holocentridae														
*Myripristis adusta*	Pk		X	X										
*Myripristis berndti*	Pk	X	X	X	X									
*Myripristis chryseres*	Pk									X	X	X	X	
*Myripristis kuntee*	Pk	X	X	X	X	X	X	X	X	X	X			
*Myripristis murdjan*	Pk		X											
*Myripristis violacea*	Pk	X	X	X										
*Myripristis vittata*	Pk					X	X	X	X	X	X			
*Neoniphon argenteus*	MIF			X										
*Neoniphon aurolineatus*	MIF						X	X	X	X	X	X		
*Neoniphon opercularis*	MIF	X	X	X										
*Sargocentron caudimaculatum*	MIF		X	X	X									
*Sargocentron microstoma*	MIF	X	X	X										
*Sargocentron spiniferum*	MIF	X												
*Sargocentron tiereoides*	MIF	X	X	X	X	X	X	X	X	X	X			
*Sargocentron tiere*	MIF	X												
*Sargocentron violaceum*	MIF	X	X	X	X	X	X	X	X	X	X			
Aulostomidae														
*Aulostomus chinensis;*	Ps	X												
Scorpaenidae														
*Pterois antennata*	MIF						X							
Serranidae														
*Aethaloperca rogaa*	Ps				X	X								
*Anyperodon leucogrammicus*	Ps	X	X	X	X	X								
*Belonoperca chabanaudi*	MIF		X											
*Cephalopholis argus*	Ps	X	X	X										
*Cephalopholis aurantia*	Ps					X	X	X	X	X	X	X		
*Cephalopholis leopardus*	Ps	X												
*Cephalopholis polleni*	Ps					X	X	X	X	X	X			
*Cephalopholis sexmaculata*	Ps	X												
*Cephalopholis spiloparaea*	Ps	X	X	X	X	X	X	X	X	X	X	X		
*Cephalopholis urodeta*	Ps	X												
*Epinephelus maculatus*	Ps										X			
*Epinephelus merra*	Ps			X										
*Epinephelus polyphekadion*	Ps	X	X	X	X	X								
*Epinephelus spilotoceps*	Ps	X												
*Gracila albomarginata*	Ps	X	X	X	X	X	X							
*Plectropomus areolatus*	Ps		X											
Serranidae, subfamily Anthiadinae														
*Luzonichthys seaver*	Pk									X	X			
*Odontanthias borbonius*	Pk											X	X	X
*Pseudanthias cooperi*	Pk					X								
*Pseudanthias dispar*	Pk	X												
*Pseudanthias flavoguttatus*	Pk									X	X	X	X	
*Pseudanthias* undescribed sp.	Pk											X		
*Pseudanthias pascalus*	Pk	X	X	X	X	X	X	X						
*Pseudanthias pleurotaenia*	Pk					X	X	X	X	X				
*Pseudanthias randalli*	Pk					X	X	X	X	X	X	X	X	
*Pseudanthias smithvanizi*	Pk			X	X	X	X	X	X	X	X	X		
*Pseudanthias ventralis*	Pk						X	X	X	X	X	X		
*Serranocirrhitus latus*	Pk					X	X							
Symphysanodontidae														
*Symphysanodon* sp.	Pk									X	X			
Cirrhitidae														
*Paracirrhites arcatus*	MIF	X	X	X										
*Paracirrhites forsteri*	Ps	X												
*Paracirrhites hemistictus*	MIF	X												
*Oxycirrhites typus*	MIF										X			
Priacanthidae														
*Priacanthus hamrur*	Pk	X	X	X	X	X								
Psuedochromidae														
*Pseudochromis cyanotaenia*	MIF	X												
Plesiopidae														
*Calloplesiops altivelis*	MIF										X	X		
Apogonidae														
*Cheilodipterus macrodon*	Pk	X	X	X	X	X	X	X	X	X	X			
*Cheilodipterus quinquelineatus*	Pk		X											
*Cheilodipterus isostigmus*	Pk					X								
*Ostorhinchus dispar*	Pk					X	X							
*Ostorhinchus nigrofasciatus*	Pk		X											
*Pristiapogon exostigma*	Pk											X	X	
Malacanthidae														
*Hoplolatilus cuniculus*	Pk									X				
*Hoplolatilus marcosi*	Pk									X	X			
*Hoplolatilus randalli*	Pk								X					
*Hoplolatilus starcki*	Pk						X							
*Malacanthus brevirostris*	MIF	X	X	X	X	X	X							
Carangidae														
*Atule mate*	Ps		X											
*Carangoides dinema*	Ps								X					
*Carangoides ferdau*	MIF	X												
*Caranx ignobilis*	Ps		X											
*Caranx lugubris*	Ps								X					
*Caranx melampygus*	Ps	X	X	X	X	X	X							
*Carangoides orthogrammus*	MIF	X	X											
*Carangoides plagiotaenia*	Ps	X												
*Caranx sexfasciatus*	Ps		X											
*Gnathanodon speciosus*	MIF	X	X											
*Trachinotus blochii*	SIF													
Lutjanidae														
*Aphareus furca*	Ps	X												
*Lutjanus decussatus*	MIF	X	X	X	X	X								
*Lutjanus ehrenbergii*	MIF		X											
*Lutjanus fulvus*	MIF				X	X								
*Lutjanus bohar*	Ps	X	X	X	X	X	X	X	X					
*Lutjanus kasmira*	MIF						X	X						
*Lutjanus semicinctus*	Ps	X	X	X										
*Macolor macularis*	Ps	X	X	X	X	X								
*Paracaesio sordida*	Pk										X	X		
Lutjanidae subfamily Caesioninae														
*Caesio caerulaurea*	Ps				X	X	X							
*Caesio teres*	Ps						X							
*Pterocaesio pisang*	Pk				X	X	X							
*Pterocaesio tile*	Pk	X	X	X	X	X								
Haemulidae														
*Plectorhinchus lineatus*	MIF				X	X								
Lethrinidae														
*Gnathodentex aureolineatus*	MIF	X	X	X										
*Lethrinus xanthochilus*	MIF	X	X											
*Monotaxis grandoculis*	MIF	X	X	X										
Mullidae														
*Parupeneus barberinus*	MIF	X	X											
*Parupeneus crassilabris*	MIF				X									
*Parupeneus cyclostomus*	Ps	X	X	X	X									
*Parupeneus multifasciatus*	MIF	X	X	X	X									
*Parupeneus trifasciatus*	MIF	X												
Pempheridae														
*Pempheris oualensis*	Pk	X												
Kyphosidae														
*Kyphosus cinerascens*	H	X	X											
*Kyphosus vaigiensis*	H		X											
Chaetodontidae														
*Chaetodon auriga*	C	X	X	X	X	X								
*Chaetodon bennetti*	C	X	X	X	X	X								
*Chaetodon burgessi*	Pk								X	X	X	X	X	X
*Chaetodon citrinellus*	C	X												
*Chaetodon ephippium*	C	X	X	X	X									
*Chaetodon kleinii*	C	X	X	X	X	X	X	X	X	X				
*Chaetodon lineolatus*	C				X									
*Chaetodon lunulatus*	C	X	X	X	X	X	X							
*Chaetodon mertensii*	MIF	X	X	X	X	X	X	X	X	X	X			
*Chaetodon ornatissimus*	C	X	X											
*Chaetodon punctatofasciatus*	C	X	X	X	X									
*Chaetodon rafflesi*	C	X												
*Chaetodon reticulatus*	C	X	X											
*Chaetodon semeion*	C	X	X	X										
*Chaetodon trifascialis*	C	X												
*Chaetodon ulietensis*	C	X	X	X	X									
*Chaetodon vagabundus*	SIF	X												
*Forcipiger flavissimus*	MIF	X	X	X	X	X	X	X	X	X	X	X		
*Forcipiger longirostris*	MIF	X	X	X	X	X	X	X	X	X	X	X	X	X
*Hemitaurichthys polylepis*	Pk	X	X	X	X	X	X							
*Heniochus acuminatus*	Pk										X	X		
*Heniochus chrysostomus*	Pk		X	X	X	X	X							
*Heniochus singularius*	C		X											
*Heniochus varius*	C	X												
Pomacanthidae														
*Apolemichthys griffisi*	SIF					X	X	X	X	X	X	X		
*Centropyge aurantia*	H		X											
*Centropyge bispinosa*	H	X	X	X	X									
*Centropyge colini*	H							X	X	X	X	X		
*Centropyge flavissima*	H	X	X	X										
*Centropyge heraldi*	H			X	X	X								
*Centropyge loriculus*	H	X	X	X	X									
*Centropyge multicolor*	H				X	X	X	X	X	X	X			
*Centropyge vroliki*	H	X	X	X										
*Genicanthus bellus*	Pk					X	X	X	X	X	X	X		
*Genicanthus watanabei*	Pk							X	X					
*Paracentropyge multifasciata*	H			X	X	X	X	X	X	X	X			
*Pomacanthus imperator*	SIF			X			X							
*Pygoplites diacanthus*	SIF	X	X	X	X	X	X	X	X	X	X	X		
Pomacentridae														
*Abudefduf septemfasciatus*	H		X											
*Abudefduf vaigiensis*	Pk	X												
*Amblyglyphidodon aureus*	Pk	X	X	X	X	X	X							
*Amphiprion chrysopterus*	Ps	X	X	X	X									
*Amphiprion clarkii*	Ps						X	X						
*Amphiprion perideraion*	Ps		X											
*Chromis acares*	Pk	X												
*Chromis agilis*	Pk		X	X	X	X	X	X	X	X	X	X	X	
*Chromis alpha*	Pk		X	X	X	X	X	X	X	X	X	X		
*Chromis amboinensis*	Pk		X											
*Chromis atripes*	Pk		X											
*Chromis brevirostris*	Pk								X	X	X	X		
*Chromis caudalis*	Pk					X								
*Chromis circumaurea*	Pk												X	X
*Chromis degruyi*	Pk									X	X			
*Chromis delta*	Pk		X											
*Chromis margaritifer*	Pk	X	X	X										
*Chromis ternatensis*	Pk	X	X	X	X	X	X	X	X					
*Chromis vanderbilti*	Pk	X												
*Chromis xanthura*	Pk	X	X											
*Chrysiptera brownriggii*	H	X												
*Chrysiptera caeruleolineata*	Pk				X									
*Chrysiptera oxycephala*	Pk		X											
*Chrysiptera traceyi*	Pk	X												
*Dascyllus trimaculatus*	Pk	X	X											
*Plectroglyphidodon dickii*	H	X	X											
*Plectroglyphidodon johnstonianus*	C	X	X	X										
*Plectroglyphidodon lacrymatus*	H	X	X	X										
*Pomacentrus auriventris*	Pk	X												
*Pomacentrus coelestis*	Pk		X											
*Pomacentrus moluccensis*	H					X	X							
*Pomacentrus philippinus*	Pk		X											
*Pomacentrus vaiuli*	H	X	X											
*Stegastes fasciolatus*	H	X												
Labridae														
*Anampses melanurus*	MIF										X	X		
*Bodianus anthioides*	MIF						X	X	X					
*Bodianus bimaculatus*	MIF										X			
*Bodianus dictynna*	MIF						X	X						
*Bodianus mesothorax*	MIF	X	X	X	X	X	X							
*Cheilinus fasciatus*	MIF		X	X	X	X	X	X	X					
*Cheilinus oxycephalus*	MIF	X	X											
*Cheilinus undulatus*	MIF	X	X	X	X	X	X							
*Cirrhilabrus earlei*	Pk									X	X	X		
*Cirrhilabrus katherinae*	Pk			X	X	X								
*Cirrhilabrus rhomboidalis*	Pk									X	X			
*Coris gaimard*	MIF	X	X	X	X									
*Epibulus insidiator*	MIF	X	X	X	X	X								
*Gomphosus varius*	MIF	X	X	X										
*Halichoeres biocellatus*	MIF			X										
*Halichoeres chrysus*	MIF		X											
*Halichoeres hartzfeldii*	MIF		X	X										
*Halichoeres hortulanus*	MIF	X	X	X	X									
*Halichoeres marginatus*	MIF	X	X											
*Halichoeres melasmapomus*	MIF		X	X	X									
*Hemigymnus fasciatus*	MIF	X	X											
*Hemigymnus melapterus*	MIF	X												
*Labroides bicolor*	MIF	X												
*Labroides dimidiatus*	MIF	X	X	X	X	X	X	X	X	X	X			
*Labropsis micronesica*	C	X												
*Labroides pectoralis*	MIF	X	X	X										
*Labrichthys unilineatus*	C	X												
*Labropsis xanthonota*	C	X												
*Macropharyngodon meleagris*	SIF	X	X	X	X	X	X							
*Novaculops halsteadi*	MIF									X	X			
*Oxycheilinus arenatus*	Ps		X	X	X	X	X	X	X	X				
*Oxycheilinus bimaculatus*	Ps												X	
*Oxycheilinus orientalis*	Ps	X	X	X	X									
*Oxycheilinus unifasciatus*	Ps	X												
*Pseudocheilinus evanidus*	MIF	X	X	X	X	X	X							
*Pseudocheilinus hexataenia*	MIF		X	X										
*Pseudocheilinus ocellatus*	MIF							X	X	X	X			
*Pseudocoris yamashiroi*	Pk		X						X	X				
*Pseudocheilinus octotaenia*	MIF	X	X	X	X									
*Stethojulis bandanensis*	MIF	X												
*Terelabrus rubrovittatus*	Ps									X	X	X	X	
*Thalassoma amblycephalum*	Pk	X												
*Thalassoma lutescens*	MIF	X	X	X										
*Thalassoma quinquevittatum*	MIF	X												
*Wetmorella nigropinnata*	MIF	X	X	X										
Labridae, subfamily Scarinae														
*Bolbometopon muricatum*	H	X	X											
*Calotomus carolinus*	H			X										
*Cetoscarus ocellatus*	H	X												
*Chlorurus bleekeri*	H	X												
*Chlorurus japanensis*	H	X												
*Chlorurus microrhinos*	H	X	X											
*Chlorurus sordidus*	H	X	X	X	X									
*Hipposcarus longiceps*	H	X												
*Scarus festivus*	H	X												
*Scarus frenatus*	H	X												
*Scarus fuscocaudalis*	H				X									
*Scarus niger*	H	X	X	X										
*Scarus oviceps*	H	X												
*Scarus rubroviolaceus*	H		X											
*Scarus schlegeli*	H		X											
*Scarus spinus*	H	X	X	X										
Pinguipedidae														
*Parapercis clathrata*	MIF	X	X	X	X	X	X	X	X	X	X			
*Parapercis schauinslandii*	MIF									X	X			
Blennidae														
*Cirripectes variolosus*	H	X												
*Meiacanthus atrodorsalis*	Pk	X	X	X										
*Plagiotremus laudandus*	Ps^*^	X												
*Plagiotremus tapeinosoma*	Ps^*^	X												
Gobiidae														
*Nemateleotris decora*	Pk									X	X			
*Nemateleotris helfrichi*	Pk	X	X	X	X	X	X							
*Nemateleotris magnifica*	Pk	X												
*Ptereleotris evides*	Pk	X	X	X										
*Ptereleotris heteroptera*	Pk				X	X	X	X	X					
*Tryssogobius* sp.	MIF										X			
Ephippidae														
*Platax orbicularis*	H	X	X	X										
Zanclidae														
*Zanclus cornutus*	SIF	X	X	X	X	X	X							
Acanthuridae														
*Acanthurus guttatus*	H	X												
*Acanthurus leucocheilus*	H	X	X											
*Acanthurus lineatus*	H	X												
*Acanthurus nigricauda*	H	X												
*Acanthurus nigrofuscus*	H		X											
*Acanthurus nigricans*	H	X	X	X	X	X	X							
*Acanthurus nigros*	H			X										
*Acanthurus pyroferus*	H	X	X	X	X	X								
*Acanthurus thompsoni*	Pk	X	X	X	X	X	X							
*Acanthurus triostegus*	H	X	X											
*Ctenochaetus binotatus*	D	X	X	X	X	X	X							
*Ctenochaetus cyanocheilus*	D		X	X	X	X	X							
*Ctenochaetus hawaiiensis*	D	X	X	X	X	X								
*Ctenochaetus striatus*	D	X	X	X	X	X	X							
*Ctenochaetus tominiensis*	D	X	X											
*Naso brevirostris*	Pk	X												
*Naso hexacanthus*	Pk	X	X	X	X	X	X							
*Naso lituratus*	Pk	X	X	X	X	X	X							
*Naso unicornis*	Pk	X	X	X	X	X	X							
*Zebrasoma scopas*	H	X	X	X	X	X								
*Zebrasoma veliferum*	H	X	X	X	X									
Siganidae														
*Siganus argenteus*	H	X												
*Siganus doliatus*	H	X												
*Siganus vulpinus*	H	X	X	X										
Sphyraenidae														
*Sphyraena barracuda*	Ps	X												
*Sphyraena qenie*	Ps	X	X	X										
Scombridae														
*Gymnosarda unicolor*	Ps	X	X	X	X	X								
Balistidae														
*Balistoides conspicillum*	MIF				X	X	X							
*Balistapus undulatus*	MIF	X	X	X	X	X	X							
*Balistoides viridescens*	MIF	X	X	X	X	X	X							
*Melichthys niger*	H	X	X	X										
*Melichthys vidua*	H	X	X											
*Odonus niger*	Pk					X	X	X	X	X	X	X		
*Pseudobalistes flavimarginatus*	MIF		X	X	X	X	X	X	X					
*Rhinecanthus rectangulus*	MIF	X												
*Sufflamen bursa*	MIF	X	X	X	X	X	X	X	X	X	X	X	X	
*Sufflamen chrysopterum*	MIF	X												
*Sufflamen fraenatum*	MIF									X				
*Xanthichthys caeruleolineatus*	Pk										X			
Monacanthidae														
*Amanses scopas*	C	X												
*Cantherhines dumerilii*	C	X												
*Cantherhines pardalis*	SIF	X												
Ostraciidae														
*Ostracion meleagris*	SIF	X	X											
Tetraodontidae														
*Arothron nigropunctatus*	C	X	X	X	X	X	X							
*Canthigaster epilampra*	MIF										X			
*Canthigaster leoparda*	SIF									X	X			

**Notes.**

Trophic guild designation C corallivore H herbivore MIF mobile invertebrate feeder Ps piscivore Pk planktivore SIF sessile invertebrate feeder

* Members of the genus *Plagiotremus* are specialized scale eaters and have been grouped with piscivores.

The results of the sample-based rarefaction curves using Michaelis–Menten richness estimators (Fig. S1) indicated that a large sampling effort (approximately 60–80 surveys per depth) would be required to completely characterize the fish communities at 10–40 m depths, whereas approximately 25 surveys would have been required to sample a complete representation of the community at 50–60 m depths. The total number of species surveyed per depth and nonparametric species richness estimators are summarized in Table S4. Species richness estimators indicate a higher number of species at depth compare to what we recorded. The highest number of species recorded was at 30 m (*n* = 73) and is the depth with the highest expected number of species based on ACE and Chao1 estimates (101–107). The lowest number of species recorded (*n* = 52) was at 40 m and is also the depth with the lowest estimated number of species (64–65).

**Figure 2:**
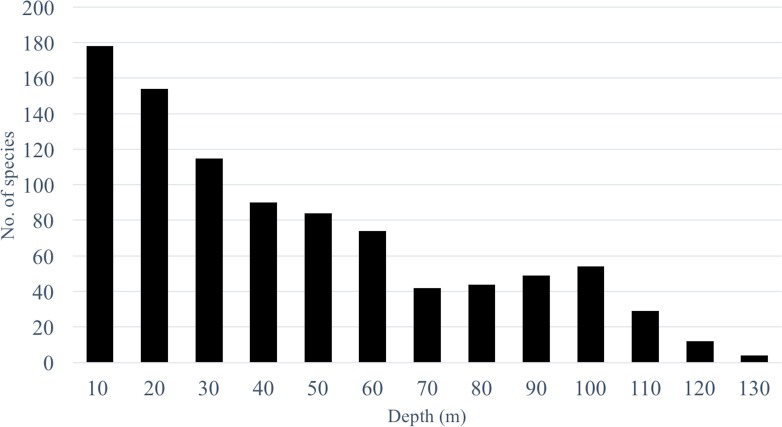
Species richness across a depth gradient in Pohnpei, FSM. Although presented as a single digit, depth was assessed across the full 10 meter increment (i.e., 10, 0–10 m; 20, 11–20 m; and so forth).

Species richness from the video survey analysis ranged from 178 species at 10 m to 4 species at 130 m (Fig. 2) and showed a steady decline after 10 m with a sharp drop between 60 and 70 m where species richness declined from 74 to 41 species. Below 70 m we observed a gradual increase to 54 species at 100 m and another sharp decrease to 29 species at 110 m. It should be noted that as depth increased, our sampling effort also decreased. Therefore, species richness estimates at deeper depths are underestimated and should be interpreted with caution.

### Comparisons across depth gradient based on transects surveys

**Figure 3:**
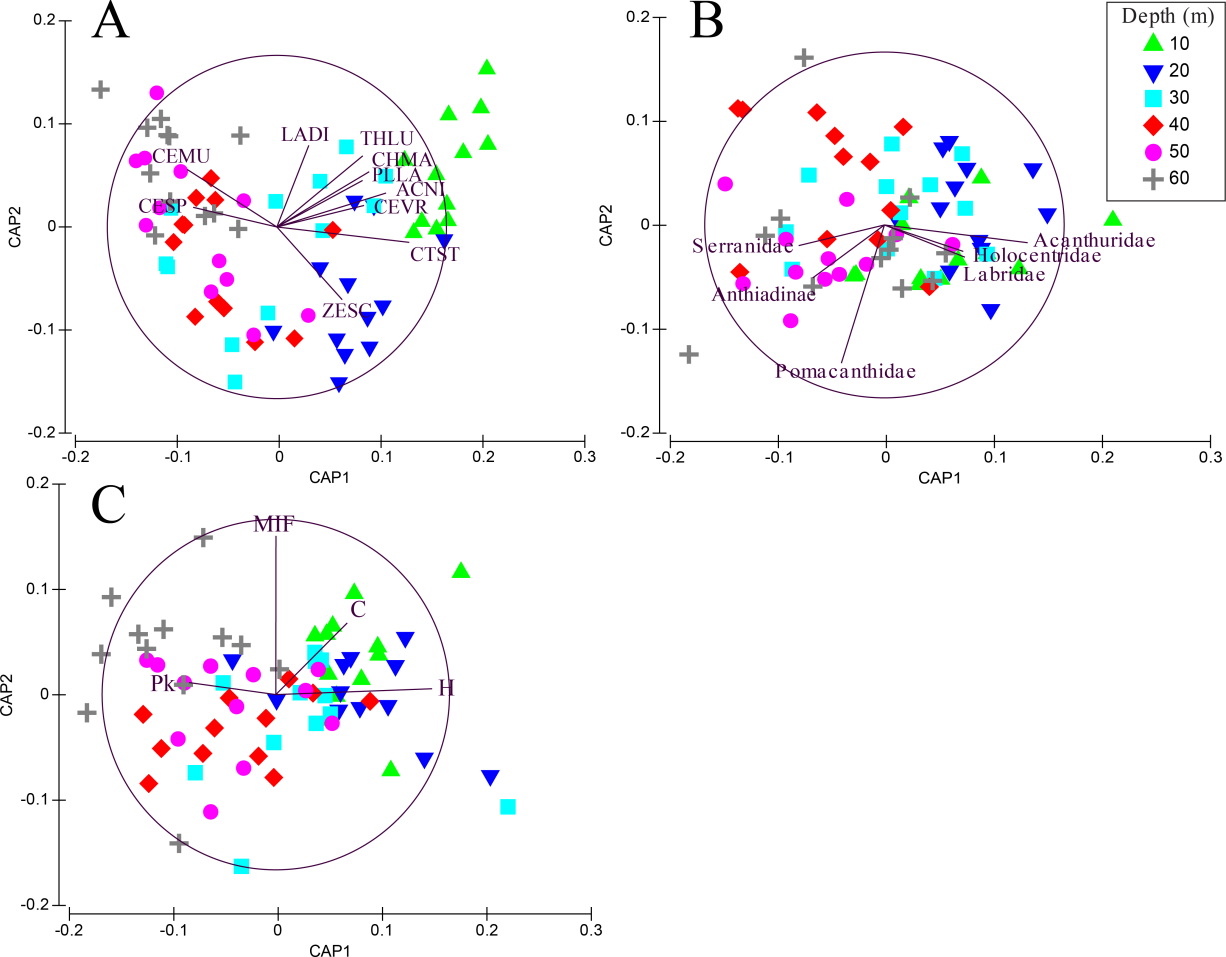
CAP summary. Canonical analysis of principal components (CAP). This is a two-dimensional representation of the multivariate space of each biological or ecological variable, (A) species (species codes: ACNI, *Acanthurus nigricans*; CEMU, *Centropyge multicolor*; CEVR, *Centropyge vroliki*; CESP, *Cephalopholis spiloparaea*; CHMA, *Chromis margaritifer*; CTST, *Ctenochaetus striatus*; LADI, *Labroides dimidiatus*; PLLA, *Plectroglyphidodon lacrymatus*; THLU, *Thalassoma lutescens*; ZESC, *Zebrasoma scopas)*; (B) family; (C) trophic guild (trophic code: C, corallivores; H, herbivores; Pk, planktivores; MIF, mobile invertebrate feeder), as it relates to depth. Points that are closer together are more similar than points that are further apart; the direction of the line indicates which depth the variable is correlated relative to the axes, and the length of the vector relative to the radius of the circle shows the correlative strength between the variable and the CAP axes. Crosses indicate Spearman’s correlation at >0.5. See inset for depth key.

Transects revealed significant differences among depths when fish were grouped by species (PERMANOVA, Pseudo-*F* =3.01, *P* < 0.001. After controlling for false discovery rates (corrected *α* = 0.015), the species assemblage at 10 m was significantly different from all other depths (Table 2). Species found at 20 m were different from all depths except 30 m. Species assemblages at 30–60 m did not differ significantly. The CAP analysis revealed the similarity in shallow assemblages is correlated with the abundance of several shallow water species such as *Thalassoma lutescens, Centropyge vroliki, Plectroglyphidodon lacrymatus, Chromis margaritifer,* as well as a generalist species *Ctenochaetus striatus* (Fig. 3A). *C. striatus* was also found to be the largest contributor (35.6–41.2%) between 10–20 m (Table S5). The similarity across 40–60 m depths is correlated with the abundance of *Centropyge multicolor* and *Cephalopholis spiloparaea* based on our CAP analysis despite *Chromis ternatensis* and *C. alpha* being the largest contributors at these depths. PERMDISP pairwise comparisons did not show a clear pattern of significance for species nor for family or trophic level comparisons indicating dispersion between depths was not a driving factor for these patterns.

**Table 2:** PERMANOVA and PERMDISP. Permutational analysis of variance (PERMANOVA) *p*-values and permutational analysis of dispersions (PERMDISP) *p*-values, obtained using 9,999 permutations, for pairwise fish assemblage comparisons between samples from depths of 10–60 m. Italicized numbers indicate significance at *p* < 0.05. Bolded numbers indicate significance after correcting for false discovery rates (corrected *α* = 0.015).

Depth (m) comparisons	Species			Family			Trophic		
	PERMANOVA		PERMDISP	PERMANOVA		PERMDISP	PERMANOVA		PERMDISP
	*p*-value	Unique permutations	*p*-value	*p*-value	Unique permutations	*p*-value	*p*-value	Unique permutations	*p*-value
10, 20	**0.0016**	9,869	0.301	0.2165	9,998	0.252	0.5611	9,904	0.243
10, 30	**0.0001**	9,855	*0.019*	0.064	9,984	0.162	**0.0157**	9,917	0.199
10, 40	**0.0001**	9,861	0.533	**0.0003**	9,905	**0.032**	**0.0001**	9,919	0.063
10, 50	**0.0001**	9,871	**0.004**	**0.0002**	9,900	0.086	**0.0001**	9,924	0.155
10, 60	**0.0001**	9,855	**0.001**	**0.0027**	9,891	*0.015*	**0.0001**	9,922	0.137
20. 30	*0.0325*	9,859	0.17	0.0855	9,910	0.955	0.4968	9,915	0.750
20. 40	**0.0003**	9,863	0.888	**0.0006**	9,903	0.368	**0.0026**	9,918	0.516
20. 50	**0.0001**	9,855	0.283	**0.0001**	9,888	0.545	**0.0007**	9,915	0.873
20, 60	**0.0001**	9,878	*0.019*	**0.0075**	9,832	0.132	**0.0001**	9,931	0.951
30, 40	0.6761	9,851	0.169	0.3369	9,895	0.372	0.3518	9,938	0.669
30, 50	0.2866	9,847	0.752	*0.0212*	9,897	0.519	*0.0385*	9,923	0.854
30, 60	0.9064	9,872	0.583	0.5028	9,903	0.112	**0.0001**	9,930	0.799
40, 50	0.6988	9,877	0.297	0.1677	9,904	0.675	0.6702	9,918	0.582
40, 60	0.0505	9,890	*0.047*	0.3263	9,892	0.809	*0.0307*	9,914	0.471
50, 60	0.3547	9,873	0.355	0.2762	9,883	0.415	**0.0051**	9,937	0.925

**Notes.**

Resemblance matrices created for PERMANOVA and PERMDISP were based on the Bray–Curtis similarity index.

**Figure 4:**
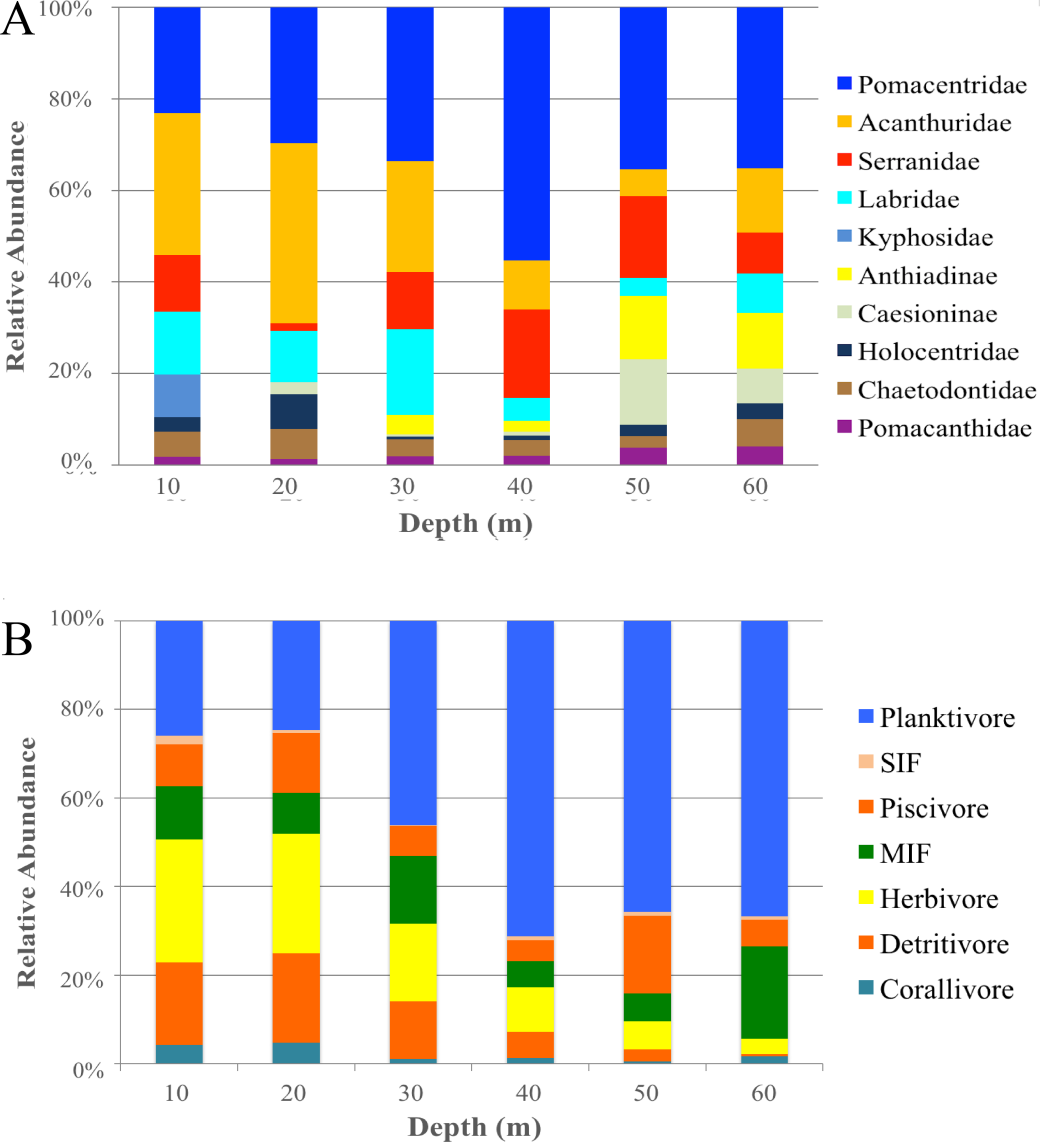
Family and trophic guild summary. Relative abundance of the 10 most common families (A) and trophic guilds (B) of reef fishes across depths at Pohnpei, FSM. Serranidae includes groupers and soapfishes.

When grouping fish by family there was a significant difference in assemblages between depths (PERMANOVA, Pseudo-*F* = 2.24 , *P* < 0.001). There were no significant differences among shallow communities (10–30 m) or among deep communities (30–60 m) but significant differences existed between the shallow and deep communities (Table 2). Family assemblages at 30 m were not significantly different from any depths. Serranids (including subfamily Anthiadinae) and pomacentrids were correlated with deep depths and acanthurids, holocentrids and labrids were correlated with shallower depths (Fig. 3B). Acanthurids were observed at all depths but had a higher relative abundance at shallow depths (10–30 m) where they made up 25–36% of the community (Fig. 4A). Despite their decline in relative abundance as depth increased, acanthurids continued to be one of the dominate contributors to the community throughout all depths with the exception of 50 m (Table S5). Pomacentrids made up a large portion of the community across all surveyed depths (22.3–51.0%). Anthiadines (Anthiadinae) were absent from 10 and 20 m and observed at higher proportions at 50 and 60 m. Fusiliers (Caesionidae) were less than 2% of the community at 10–40 m but increased to 14% and 18% at 50 and 60 m, respectively. Labrids had their highest relative abundance between 10–30 m (10–19%), were less abundant at 40 and 50 m (4–5%) and then increased at 60 m (up to 9%). Non-anthiandinae serranids only made up 1% of the community at 20 m but had relatively high abundance at all other depths (9–20%).

The highest contributors across all depths were consistently herbivores (with the exception of 60 m), planktivores and mobile invertebrate feeders (Table S5). Although, trophic guilds were similar between 10–30 m and 30–50 m, these two groups were significantly different from each other indicating two distinct trophic regimes that overlapped at 30 m (Table 2). Assemblages at 60 m were significantly different from all other depths except 40 m and assemblages at 30 m were similar to all other depths. The shallow trophic guilds were dominated by corallivores and herbivores while the deeper depths were dominated by planktivores (Fig. 3C). The relative abundance of trophic guilds across depths showed herbivores dominating the shallow depths (10 and 20 m, Fig. 4B) making up 46–47% of the community, a finding corroborated by our SIMPER analysis which showed herbivores being the largest contributor at these depths (39% and 45% at 10 and 20 m, respectively). Although corallivores were found throughout all depths, their highest relative abundance was at shallow depths (10 and 20 m). Planktivores began to dominate at 40 m, making up 71% of the community where they became the largest contributor, a pattern that continued to 60 m (55%). At 60 m, herbivores and corallivores made up less than 10% of the community. Sessile invertebrate feeders were found at all depths but made up no more than 2% of the community at each depth. The highest relative abundance of piscivores was observed at 50 m, with 18% of the community. Mobile invertebrate feeders had their highest proportion (21%) and become one of the dominate contributors (29%) at 60 m with their lowest proportions (6%) at 40 and 50 m.

### Comparisons across depth gradient based on video surveys

**Figure 5:**
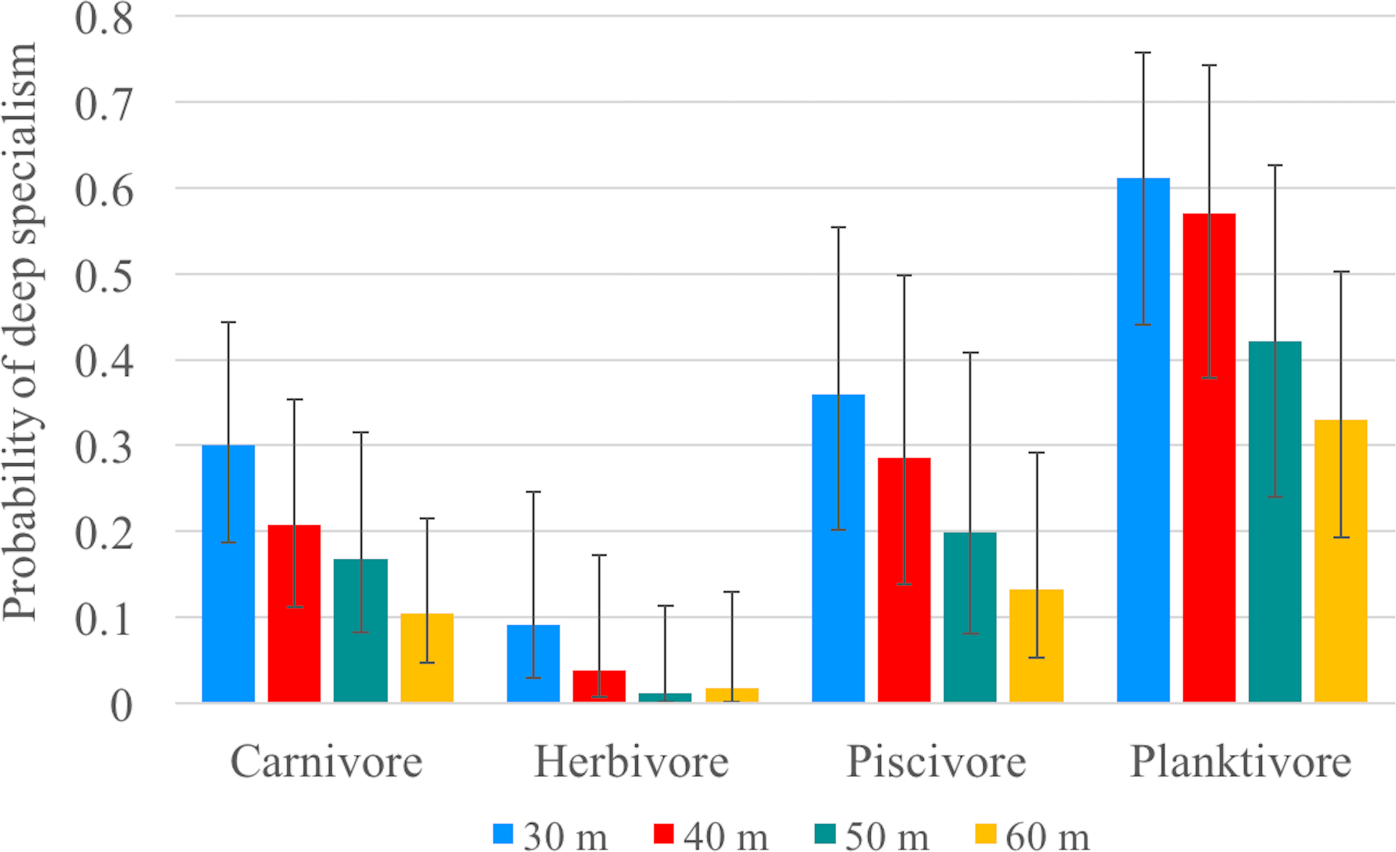
Specialism probability (GLMM). Mean probability of being classified as a deep specialist for all trophic guilds according to generalized linear mixed-effects models based on each specified depth contour (color). Error bars represent 95% confidence limits.

Our video surveys showed trends similar to our transect surveys among trophic guilds for models using 30, 40, 50 and 60 m depth contours as the transition from ‘shallow specialists’ to ‘deep specialists’. All models were significantly different from the null model (*p* < 0.001) (Table 3). For all four models, herbivores exhibited significantly shallower depth ranges (except when compared to piscivores in the 50 m model) and planktivores exhibited significantly deeper depth ranges (except when compared to piscivores in the 50 and 60 m models) compared to all other trophic guilds (Fig. 5). There was no significant difference between carnivores and piscivores across all four models. The models explained 8–9% of deviance in whether or not a species was classified as a deep specialist indicating there are additional factors beyond our model explaining a significant portion of depth distribution (Table S6). The bulk of species diversity found at Pohnpei is comprised of shallow specialists that accounted for 47–73% of the diversity depending on the specified depth contour. Deep specialists accounted for 14–25% of the diversity while depth-generalists ranged from 13–27%.

**Table 3:** GLMM. Generalized linear mixed-effect model results on the effect of trophic guild on deep specialists according to each depth contour. Bolded values indicate significance at *P* < 0.05. Reference level for trophic guild is set as ‘Carnivore’. S.E., standard error.

	Estimate ± S.E.	*z*-value	*p*-value
30 m threshold			
Intercept	**−0.824 ± 0.320**	**−2.575**	**0.010**
Herbivores	**−1.463 ± 0.668**	**−2.191**	**0.028**
Piscivores	0.176 ± 0.494	0.357	0.721
Planktivores	**1.276 ± 0.444**	**2.873**	**0.004**
40 m threshold			
Intercept	**−1.236 ± 0.361**	**−3.432**	**0.001**
Herbivores	**−1.944 ± 0.878**	**−2.213**	**0.027**
Piscivores	0.255 ± 0.532	0.479	0.632
Planktivores	**1.539 ± 0.493**	**3.123**	**0.002**
50 m threshold			
Intercept	**−1.497 ± 0.397**	**−3.773**	**0.000**
Herbivores	**−2.788 ± 1.176**	**−2.372**	**0.018**
Piscivores	0.050 ± 0.590	0.084	0.933
Planktivores	**1.184 ± 0.547**	**2.165**	**0.030**
60 m threshold			
Intercept	**−2.047 ± 0.409**	**−5.005**	**<0.001**
Herbivores	−1.915 ± 1.123	−1.705	0.088
Piscivores	0.238 ± 0.575	0.413	0.679
Planktivores	**1.343 ± 0.514**	**2.614**	**0.009**

## Discussion

### Transition zone between shallow and mesophotic fish communities

Investigations of fish communities associated with MCEs have primarily focused on the Caribbean, South Atlantic Ocean, Red Sea, and Hawai’i (Bridge et al., 2016; Brokovich et al., 2010; De Oliveira Soares et al., 2016; Kahng et al., 2010; Pinheiro et al., 2016; Pyle et al., 2016; Simon et al., 2016). Here, we characterize the fish community along a shallow to mesophotic depth gradient in the central Pacific Ocean. Our results based on species abundance and presence/absence highlight a difference in fish community assemblages between 30 and 60 m (Tables 2, 3) with a transition from shallow to mesophotic assemblages at 30 m, a pattern consistent with previous studies (Bejarano, Appeldoorn & Nemeth, 2014; Brokovich et al., 2008; Garcia-Sais, 2010; Kane & Tissot, 2017; Rosa et al., 2016). Additionally, we observed a shift among taxonomic families as depth increases with acanthurids, holocentrids, and labrids associated primarily with shallow habitat, while pomacentrids and serranids (including anthiadines) were more abundant at deeper depths (Figs. 3B, 4A). These results contrast with Brokovich et al. (2008) who documented an increasing Labridae-dominated community from 40–70 m, indicating that trends based on taxonomic families may vary between regions or by microhabitat type within MCEs.

By increasing our surveys to 130 m we found preliminary evidence of multiple transitional depths. Starting at 70 m we observed an unexpected increase in species richness peaking at 100 m (*n* = 54) species preceding a sharp decline at 110 m (*n* = 29) (Fig. 2). This trend follows the predictions of the mid-domain effect that predicts a peak in species richness due to an overlap in species ranges towards the center of a shared, bounded domain (Colwell & Hurtt, 1994). It has only been recently that transect surveys of MCEs have included depths deeper than 60 m (Fukunaga et al. 2016; Kane & Tissot 2017; Pinheiro et al. 2016; Rosa et al. 2016) which may explain why this trend has not been previously identified. To determine whether this trend is a regional anomaly or a global pattern, and to identify the mechanism facilitating this trend, further studies that incorporate depths >60 m are required.

### Factors influencing depth distributions of fishes

Since changing depths presents both physiological and ecological challenges, perhaps community-depth relationships align with (trophic) functional groups more than taxonomic groups. Trophic position has been previously identified as a predictor of mesophotic fish assemblages (Bridge et al., 2016). A recent study by Kane & Tissot (2017) in Hawai’i concluded that trophic position accounts for 31% of the variation in community composition between shallow and mesophotic environments. Food availability likely drives vertical distribution, particularly for species with light-dependent food sources. As expected, herbivorous fishes dominated shallow depths and had a low probability of being a deep specialist. Our model using a 30-m depth contour predicted a <10% mean probability that an herbivore will be a deep specialist, which decreases to 1% when using a 50- or 60-m depth contour (Fig. 5). Of the 41 herbivorous species, only *Centropyge colini* was observed deeper than 30 m, appearing at 70–110 m in our study. While classified as an herbivore (Allen, Steene & Allen, 1998), *C. colini* is an understudied species due its secretive nature and deep habitat; therefore, its natural diet may extend beyond herbivory. Despite low light intensity and a limited spectrum at mesophotic depths (Lesser, Slattery & Leichter, 2009; Mass et al., 2007), algal communities thrive as deep as 268 m providing potential habitat for herbivores. However, the algal community changes drastically along the depth gradient (Littler et al., 1985) with a decrease in algal grazing pressure as depth increases (Brokovich et al., 2010). No macroalgae were observed deeper than 30 m during our surveys and the filamentous mesophotic algal community of Pohnpei is currently unknown.

The abundance of corallivores also declined beyond 20 m. Most scleractinian corals are found on the continental shelf where there are known to thrive as deep as 153 m (Kahng & Maragos, 2006), but can even occur at abyssal depths as deep as 6,328 m (Keller, Oskina & Savilova, 2017). Despite this ability to inhabit deep zones, none were not observed during our deep surveys, indicating that corallivorous species may also be limited by food availability at depth. In contrast, planktivores are not light dependent and this was the only trophic category observed across all depths. Planktivores had the highest relative abundance and planktivory was the dominant trophic position at deeper depths, a pattern observed elsewhere in the Pacific (Pyle et al., 2016). The mean probability of a planktivore being a deep specialist was as high as 61% using a 30-m depth contour (Fig. 5), indicating that planktivory is the dominant feeding strategy on mesophotic reefs. It has been hypothesized that planktivory on MCEs is the result of increased nutrient-rich water at depth (Kahng et al., 2010). An additional consideration may be reduced competition. With fewer herbivores and corallivores, there are fewer species competing for space, especially shelter.

Habitat structure is a likely factor influencing the differences between shallow and mesophotic fish assemblages observed in our study. The structural habitat created by scleractinian corals in other parts of the Pacific (Bridge et al., 2011) are not present at mesophotic depths in Pohnpei. Instead, steep slopes dominated by gorgonians and sponges characterize the MCEs of Pohnpei. Many of the fishes we observed were localized around rocky outcroppings and ledges and it may be this reduction in structural complexity and coral diversity that limits the available habitat for many shallow water fishes, particularly for those that reside within the reef itself.

Our models demonstrate that trophic position influences the depth distribution of Micronesian fishes; however, due to the low explanatory power of these models we must seek other contributing factors. Prior studies have identified differences in morphological characteristics, habitat, and indirect effects of depth itself (Bridge et al., 2016; Kane & Tissot, 2017) as factors influencing depth specialization. As MCE research progresses, so will our understanding of the ecological and physiological drivers of mesophotic specialization.

### Describing a unique mesophotic community

No global consensus exists regarding the depth at which shallow ecosystems shift to MCEs, and this likely reflects the idiosyncrasies of individual study sites. Yet our results indicate a transition beginning at 30 m from a shallow to mesophotic community, corroborating transitional depths identified in prior studies. The logistical challenges of conducting transects below 60 m prevented us from describing species abundances at deeper mesophotic sites; however, our video surveys provided the capacity to describe species present in deeper MCEs. The number of deep specialists, depending on the specified depth contour, ranged from 42 to 78 species. Garcia-Sais (2010) identified six species (*Centropyge argi*, *Prognathodes aculeatus, Chromis insolata*, *Halichoeres cyanocephalus*, *Sparisoma atomarium*, and *Xanthichthys ringens*) across five families that were indicators of Caribbean mesophotic habitat. Our results found two species with high relative abundances at mesophotic depths: *Pseudanthias pleurotaenia* and *P. cooperi* (Family Serranidae, Subfamily Anthiadinae, Table S3); however, each species has been observed above mesophotic habitat at depths as shallow as 10 and 16 m, respectively (Randall, 2005) and they are therefore not suitable as indicator species for Central Pacific mesophotic habitat. Moreover, we found 27 species that were not observed shallower than 90 m, a depth well within the accepted range of MCEs. Of these species, only four have not been observed in shallower habitat elsewhere in their range: *Chromis circumaurea, Luzonichthys seaver, Odontanthias borbonius,* and *Symphysanodon* sp. *C. circumaurea* was first reported in Yap, Marshall Islands, at 98–120 m (Pyle, Earle & Greene, 2008); *L. seaver* was recently described based on specimens collected at 90–100 m (Copus, Ka’apu-Lyons & Pyle, 2015); and *O. borbonius* is typically found at 200–300 m but has been observed as shallow as 92 m (Randall & Heemstra, 2006). A single undescribed *Symphysanodon* sp*.* specimen (R Pyle, pers. comm., 2014) was collected as part of this expedition and has yet to receive a formal description; however, other members of this genus have distributions below 150 m (Anderson & Bineesh, 2011; Anderson & Springer, 2005). Notably, all four species are planktivores. Taking into account these lines of evidence, we propose that these four species are indicators of MCEs in the Central Pacific. Additionally, *Chromis degruyi* was observed between 80–90 m in this study and the shallowest previously recorded depth is 85 m (Pyle, Earle & Greene, 2008), indicating that *C. degruyi* may also be considered an indicator species for mesophotic ecosystems.

## Conclusions

The upper boundary of mesophotic communities is likely to shift in response to changing climate, oceanography, and biotic factors. However, the emerging trend from surveys in the Caribbean, the South Atlantic Ocean, Red Sea, Hawai’i, and this Central Pacific study are remarkably consistent. Establishing 30 m as the transition zone between shallow and mesophotic fish communities in the Pacific has several implications. First, future evolutionary and ecological studies have a benchmark depth to distinguish shallow and mesophotic communities. Second, at the species, family and trophic level, there is little overlap between conspicuous diurnal reef fishes on shallow reef communities (10–20 m) and MCEs (40–60 m). Hence the utility of MCEs to act as a refuge from shallow reef disturbances (i.e., DRRH) is limited (but see Tenggardjaja, Bowen & Bernardi, 2014), a conclusion that applies to brachyuran crabs (Hurley et al., 2016) and scleractinian corals (Bongaerts et al., 2010 and references therein). Finally, the distinction between shallow and mesophotic ecosystems should be integral to the development of future management strategies. Some types of disturbances restricted to either region are not likely to immediately impact the other and both ecosystems should be considered in management of reefs near human population centers.

##  Supplemental Information

10.7717/peerj.4650/supp-1 Table S1 Survey GPS coordinates

10.7717/peerj.4650/supp-2 Table S2 Dive duration at each depth (mean ± standard deviation) across 12 dives

10.7717/peerj.4650/supp-3 Table S3 Transect survey Fish transect survey conducted at Pohnpei, Federated States of Micronesia from 10–60 meters. Numbers indicate the total number of individuals observed during each survey. Diet codes: C, corralivore; D, detritivores; H, herbivores; MIF, mobile invertebrate feeder; Pk, planktivore; Ps, piscivore; SIF, sessile invertebrate feeder.

10.7717/peerj.4650/supp-4 Table S4 Choa and ACE estimates ****Summary of observed (*S*_obs_), Abundance Coverage based Estimator (ACE), and Chao1 species richness estimators for fish assemblages at each depth.

10.7717/peerj.4650/supp-5 Table S5 SIMPER analysis Similarity percentage (SIMPER) analysis based on (a) species, (b) family and (c) trophic position indicating the variable that is contributing the highest percentage at each depth (10–60 m). Contributions up to 75% are reported. Abbreviations: Av.Abund, average abundance; Av.Sim, average similarity; Sim/SD, similarity standard deviation; Contrib%, percent contribution; Cum.%, cumulative contribution.

10.7717/peerj.4650/supp-6 Table S6 GLMM summary stats Summary statistics of the generalized linear mixed-effects models according to each depth contour. Note changes in the number of taxonomic groups reflects removal of ‘depth-generalists’ prior to analysis.

10.7717/peerj.4650/supp-7 Figure S1 Rarefaction curve Species accumulation curve from fish surveys conduced between 10–60 m on the island of Pohnpei, Federated States of Micronesia. See inset for depth key.

## Additional Information and Declarations

### Data Availability

The following information was supplied regarding data availability: The raw data for presence/absence for 0–130 m are included in Table 1.
