# Supplementary material for: Shifting reef fish assemblages along a depth gradient in Pohnpei, Micronesia
Source: PeerJ. 2018 Apr 24;6:e4650. doi: 10.7717/peerj.4650 (PMC5922234; doi:10.7717/peerj.4650)
Supplement: Table S1 [file peerj-06-4650-s001.docx]

Table S1. Coordinates of survey locations

|  |  |
| --- | --- |
| Survey Number | Coordinates |
| 1 | 6.991689, 158.136993 |
| 2 | 6.839808, 158.111206 |
| 3 | 6.783403, 158.135000 |
| 4 | 6.975783, 158.114533 |
| 5 | 6.891647, 158.095483 |
| 6 | 7.018578, 158.293941 |
| 7 | 6.790180, 158.034245 |
| 8 | 6.891667, 158.095556 |
| 9 | 6.754852, 157.920618 |
| 10 | 6.991689, 158.136993 |
| 11 | 6.991690, 158.136993 |
| 12 | 6.754722, 157.920556 |
|  |  |
