# Supplementary material for: Shifting reef fish assemblages along a depth gradient in Pohnpei, Micronesia
Source: PeerJ. 2018 Apr 24;6:e4650. doi: 10.7717/peerj.4650 (PMC5922234; doi:10.7717/peerj.4650)
Supplement: Table S2 [file peerj-06-4650-s002.docx]

**Table S2**. Dive duration at each depth (mean ± standard deviation) across 12 dives

|  |  |
| --- | --- |
| depth (m) | Time (min) |
| 0-10 | 71 ± 35 |
| 11-20 | 84 ± 49 |
| 21-30 | 17 ± 3 |
| 31-40 | 6 ± 2 |
| 41-50 | 5 ± 2 |
| 51-60 | 3 ± 1 |
| 61-70 | 3 ± 1 |
| 71-80 | 3 ± 1 |
| 81-90 | 4 ± 3 |
| 91-100 | 6 ± 4 |
| 101-110 | 3 ± 2 |
| 111-120 | 3 ± 2 |
| 121-130 | 3 ± 1 |
|  |  |
