# Supplementary material for: Shifting reef fish assemblages along a depth gradient in Pohnpei, Micronesia
Source: PeerJ. 2018 Apr 24;6:e4650. doi: 10.7717/peerj.4650 (PMC5922234; doi:10.7717/peerj.4650)
Supplement: Table S3 — Fish transect survey conducted at Pohnpei, Federated States of Micronesia from 10–60 meters. Numbers indicate the total number of individuals observed during each survey. Diet codes: C, corralivore; D, detritivores; H, herbivores; MIF, mobile invertebrate feeder; Pk, planktivore; Ps, piscivore; SIF, sessile invertebrate feeder. [file peerj-06-4650-s003.docx]

Table S3. Fish transect survey conducted at Pohnpei, Federated States of Micronesia from 10-60 meters. Numbers indicate the total number of individuals observed during each survey. Diet codes: C, corallivore; D, detritivores; H, herbivores; MIF, mobile invertebrate feeder; Pk, planktivore; Ps, piscivore; SIF, sessile invertebrate feeder

| 10 meters |  |  |  |  |  |  |  |  |  |  |  |  |  |  |
| --- | --- | --- | --- | --- | --- | --- | --- | --- | --- | --- | --- | --- | --- | --- |
|  |  |  | Survey Number | | | | | | | | | | | |
| **Species** | **Family** | **Diet** | **1** | **2** | **3** | **4** | **5** | **6** | **7** | **8** | **9** | **10** | **11** | **12** |
| *Acanthurus nigricans* | Acanthuridae | *H* | 1 |  |  | 1 | 2 | 3 | 5 | 3 | 5 | 13 | 1 | 2 |
| *Acanthurus pyroferus* | Acanthuridae | *H* |  |  |  |  | 1 |  | 1 |  |  |  |  |  |
| *Ctenochaetus binotatus* | Acanthuridae | *D* |  |  | 1 |  |  |  |  |  |  |  |  |  |
| *Ctenochaetus hawaiiensis* | Acanthuridae | *D* | 2 | 3 |  | 4 |  | 2 | 5 | 3 | 1 | 7 |  | 2 |
| *Ctenochaetus striatus* | Acanthuridae | *D* | 1 | 8 | 25 | 12 | 4 | 5 | 12 | 8 | 12 | 8 | 11 | 5 |
| *Naso lituratus* | Acanthuridae | *Pk* |  |  |  | 3 |  |  |  |  | 12 | 1 | 3 |  |
| *Zebrasoma scopas* | Acanthuridae | *H* |  | 5 | 2 |  | 3 |  |  |  |  |  |  | 1 |
| *Zebrasoma veliferum* | Acanthuridae | *H* |  |  |  | 1 |  |  |  |  |  |  | 1 | 2 |
| *Aulostomus chinensis* | Aulostomidae | *Ps* |  |  | 1 |  |  |  |  |  |  |  |  |  |
| *Balistapus undulatus* | Balistidae | *MIF* |  | 1 | 1 |  |  | 1 | 1 | 1 |  |  |  |  |
| *Melichthys vidua* | Balistidae | *H* |  |  |  |  |  |  |  |  |  |  | 1 |  |
| *Chaetodon auriga* | Chaetodontidae | *C* | 1 |  | 6 |  | 1 | 1 |  |  |  |  |  | 1 |
| *Chaetodon ephippium* | Chaetodontidae | *C* |  |  |  |  |  |  |  | 2 |  |  |  |  |
| *Chaetodon kleinii* | Chaetodontidae | *C* |  |  | 5 |  |  |  |  |  |  |  |  |  |
| *Chaetodon lunulatus* | Chaetodontidae | *C* |  | 3 |  | 2 |  |  |  |  |  |  |  |  |
| *Chaetodon punctatofasciatus* | Chaetodontidae | *C* |  |  |  |  |  |  |  |  |  |  |  | 1 |
| *Chaetodon reticulatus* | Chaetodontidae | *C* |  |  |  |  | 1 |  |  |  | 1 | 1 |  |  |
| *Chaetodon semeion* | Chaetodontidae | *C* |  | 2 |  |  |  |  |  |  |  |  |  |  |
| *Forcipiger flavissimus* | Chaetodontidae | *MIF* |  |  | 2 | 3 |  |  |  |  | 1 |  |  |  |
| *Forcipiger longirostris* | Chaetodontidae | *MIF* |  |  |  |  | 3 |  | 3 |  |  |  |  | 1 |
| *Heniochus varius* | Chaetodontidae | *C* |  |  |  |  |  |  | 1 | 2 |  |  |  |  |
| *Ptereleotris evides* | Gobiidae | *Pk* |  | 2 |  |  |  |  |  |  |  |  |  |  |
| *Myripristis kuntee* | Holocentridae | *Pk* | 6 |  | 4 |  |  |  |  | 5 | 2 | 3 |  | 1 |
| *Kyphosus cinerascens* | Kyphosidae | *H* |  |  |  |  |  |  |  |  |  |  | 6 |  |
| *Bodianus mesothorax* | Labridae | *MIF* | 1 |  |  |  |  |  |  |  |  |  |  |  |
| *Epibulus insidiator* | Labridae | *MIF* |  |  |  |  |  |  |  |  | 1 |  | 1 | 1 |
| *Gomphosus varius* | Labridae | *MIF* | 3 | 1 | 1 | 1 |  |  | 1 |  |  |  |  |  |
| *Halichoeres hartzfeldii* | Labridae | *MIF* |  |  |  |  |  | 3 |  |  |  |  |  |  |
| *Halichoeres hortulanus* | Labridae | *MIF* | 1 | 4 | 3 | 2 | 1 |  |  |  |  |  |  |  |
| *Halichoeres marginatus* | Labridae | *MIF* |  | 1 |  |  |  |  |  |  |  |  |  |  |
| *Labroides dimidiatus* | Labridae | *MIF* | 2 | 1 | 4 |  | 3 | 2 | 2 |  |  |  |  | 1 |
| *Labroides pectoralis* | Labridae | *MIF* | 1 |  |  |  |  |  |  |  |  | 1 |  |  |
| *Oxycheilinus unifasciatus* | Labridae | *Ps* |  |  |  |  |  | 1 |  |  |  |  |  |  |
| *Stethojulis bandanensis* | Labridae | *MIF* |  | 2 |  |  |  |  |  |  |  |  |  |  |
| *Thalassoma lutescens* | Labridae | *MIF* | 3 |  | 2 | 2 | 2 | 2 |  |  | 3 |  | 1 |  |
| *Chlorurus sordidus* | Labridae, subfamily Scarinae | *H* |  | 3 |  |  | 1 |  |  |  |  |  |  |  |
| *Scarus niger* | Labridae, subfamily Scarinae | *H* |  |  | 3 | 2 |  | 5 |  |  |  |  |  | 1 |
| *Scarus rubroviolaceus* | Labridae, subfamily Scarinae | *H* |  | 2 | 1 |  |  |  |  |  |  |  |  |  |
| *Scarus spinus* | Labridae, subfamily Scarinae | *H* |  |  |  |  |  |  |  |  |  |  | 1 | 1 |
| *Lutjanus semicinctus* | Lutjanidae | *Ps* |  |  |  |  |  |  |  |  |  |  | 4 |  |
| *Macolor macularis* | Lutjanidae | *Ps* |  |  |  |  |  |  | 2 |  |  |  |  |  |
| *Parupeneus cyclostomus* | Mullidae | *Ps* |  |  |  |  |  |  | 1 |  |  |  |  |  |
| *Parupeneus multifasciatus* | Mullidae | *MIF* |  | 1 | 1 |  | 1 |  | 1 |  |  |  |  |  |
| *Parupeneus trifasciatus* | Mullidae | *MIF* |  |  | 7 |  |  |  |  |  |  |  |  |  |
| *Gymnothorax flavimarginatus* | Muraenidae | *Ps* |  |  |  |  |  |  |  |  | 1 |  |  |  |
| *Centropyge flavissima* | Pomacanthidae | *H* |  |  |  |  |  | 1 |  |  |  |  |  |  |
| *Centropyge loriculus* | Pomacanthidae | *H* |  |  |  |  | 1 |  |  |  |  |  |  |  |
| *Centropyge vroliki* | Pomacanthidae | *H* |  | 2 |  | 1 |  | 2 |  | 1 |  | 1 | 1 |  |
| *Pygoplites diacanthus* | Pomacanthidae | *SIF* | 1 |  | 1 |  |  |  |  |  |  |  |  |  |
| *Amphiprion chrysopterus* | Pomacentridae | *Ps* |  |  | 2 |  | 3 |  |  |  |  |  | 2 |  |
| *Chromis acares* | Pomacentridae | *Pk* |  |  |  |  | 2 |  |  |  |  |  |  |  |
| *Chromis alpha* | Pomacentridae | *Pk* |  |  | 1 |  |  |  |  |  |  |  |  |  |
| *Chromis margaritifer* | Pomacentridae | *Pk* |  |  | 13 | 4 | 1 | 13 |  | 5 |  | 6 | 7 |  |
| *Chromis ternatensis* | Pomacentridae | *Pk* |  |  |  |  |  |  | 5 |  |  |  |  | 15 |
| *Chrysiptera brownriggii* | Pomacentridae | *H* |  | 14 | 2 |  |  |  | 2 |  |  |  |  | 5 |
| *Cirripectes variolosus* | Pomacentridae | *Pk* |  |  |  |  |  | 1 |  | 2 |  |  |  |  |
| *Dascyllus trimaculatus* | Pomacentridae | *Pk* |  |  |  |  | 1 |  |  |  |  |  | 1 |  |
| *Plectroglyphidodon lacrymatus* | Pomacentridae | *H* | 1 | 2 | 6 |  | 5 | 3 |  |  |  |  | 3 |  |
| *Pomacentrus philippinus* | Pomacentridae | *Pk* |  |  |  |  |  |  | 3 |  |  |  |  |  |
| *Pomacentrus vaiuli* | Pomacentridae | *H* |  |  |  | 1 |  |  |  |  |  |  |  |  |
| *Stegastes fasciolatus* | Pomacentridae | *H* |  |  |  |  |  |  |  |  |  |  | 2 |  |
| *Pictichromis paccagnellae* | Pseudochromidae | *Pk* | 3 |  |  |  |  |  |  |  |  |  |  |  |
| *Cephalopholis argus* | Serranidae | *Ps* |  |  |  |  | 1 |  |  |  | 1 |  |  |  |
| *Epinephelus polyphekadion* | Serranidae | *Ps* |  |  | 1 | 1 |  | 1 |  |  |  |  |  |  |
| *Pseudanthias dispar* | Serranidae, subfamily Anthiadinae | *Pk* |  |  |  |  |  | 35 |  |  |  |  |  |  |
| *Siganus vulpinus* | Siganidae | *H* |  |  |  |  |  |  |  | 1 |  |  |  |  |
| *Arothron nigropunctatus* | Tetraodontidae | *C* |  | 1 |  |  |  |  |  |  |  |  |  |  |
| *Zanclus cornutus* | Zanclidae | *SIF* |  | 1 |  | 1 |  |  |  |  |  |  | 2 |  |

| 20 meters |  |  |  |  |  |  |  |  |  |  |  |  |  |  |
| --- | --- | --- | --- | --- | --- | --- | --- | --- | --- | --- | --- | --- | --- | --- |
|  |  |  | Survey Number | | | | | | | | | | | |
| **Species** | **Family** | **Diet** | **1** | **2** | **3** | **4** | **5** | **6** | **7** | **8** | **9** | **10** | **11** | **12** |
| *Acanthurus nigricans* | Acanthuridae | *H* | 1 |  |  | 1 | 2 | 3 | 5 | 3 | 5 | 13 | 1 | 2 |
| *Acanthurus pyroferus* | Acanthuridae | *H* |  |  |  |  | 1 |  | 1 |  |  |  |  |  |
| *Ctenochaetus binotatus* | Acanthuridae | *D* |  |  | 1 |  |  |  |  |  |  |  |  |  |
| *Ctenochaetus hawaiiensis* | Acanthuridae | *D* |  |  | 2 | 3 | 1 |  |  |  |  | 7 |  | 3 |
| *Ctenochaetus striatus* | Acanthuridae | *D* | 1 | 8 | 25 | 12 | 4 | 5 | 12 | 8 | 19 | 8 | 11 | 5 |
| *Naso lituratus* | Acanthuridae | *Pk* |  |  |  | 3 |  |  |  |  | 5 | 1 | 3 |  |
| *Zebrasoma scopas* | Acanthuridae | *H* |  | 5 | 2 |  | 3 |  |  |  |  |  |  | 1 |
| *Zebrasoma veliferum* | Acanthuridae | *H* |  |  |  | 1 |  |  |  |  |  |  | 1 | 2 |
| *Aulostomus chinensis* | Aulostomidae | *Ps* |  |  | 1 |  |  |  |  |  |  |  |  |  |
| *Balistapus undulatus* | Balistidae | *MIF* |  | 1 | 1 |  |  | 1 | 1 | 1 |  |  |  |  |
| *Melichthys vidua* | Balistidae | *H* |  |  |  |  |  |  |  |  |  |  | 1 |  |
| *Meiacanthus atrodorsalis* | Blennidae | *Pk* |  |  |  |  |  | 1 |  | 2 |  |  |  |  |
| *Chaetodon auriga* | Chaetodontidae | *C* | 1 |  | 6 |  | 1 | 1 |  |  |  |  |  | 1 |
| *Chaetodon ephippium* | Chaetodontidae | *C* |  |  |  |  |  |  |  | 2 |  |  |  |  |
| *Chaetodon kleinii* | Chaetodontidae | *C* |  |  | 5 |  |  |  |  |  |  |  |  |  |
| *Chaetodon lunulatus* | Chaetodontidae | *C* |  | 3 |  | 2 |  |  |  |  |  |  |  |  |
| *Chaetodon punctatofasciatus* | Chaetodontidae | *C* |  |  |  |  |  |  |  |  |  |  |  | 1 |
| *Chaetodon reticulatus* | Chaetodontidae | *C* |  |  |  |  |  |  |  |  | 1 | 1 |  |  |
| *Chaetodon reticulatus* | Chaetodontidae | *C* |  |  |  |  | 1 |  |  |  |  |  |  |  |
| *Chaetodon semeion* | Chaetodontidae | *C* |  | 2 |  |  |  |  |  |  |  |  |  |  |
| *Forcipiger flavissimus* | Chaetodontidae | *MIF* |  |  |  |  | 3 |  | 3 |  |  |  |  | 1 |
| *Forcipiger longirostris* | Chaetodontidae | *MIF* |  |  |  |  |  |  |  |  | 2 | 2 |  | 1 |
| *Heniochus singularius* | Chaetodontidae | *C* |  |  |  |  |  |  | 1 | 2 |  |  |  |  |
| *Ptereleotris evides* | Gobiidae | *Pk* |  | 2 |  |  |  |  |  |  |  |  |  |  |
| *Myripristis kuntee* | Holocentridae | *Pk* | 6 |  | 4 |  |  |  |  | 5 | 2 | 3 |  | 1 |
| *Kyphosus cinerascens* | Kyphosidae | *H* |  |  |  |  |  |  |  |  |  |  | 6 |  |
| *Bodianus mesothorax* | Labridae | *MIF* | 1 |  |  |  |  |  |  |  |  |  |  |  |
| *Cirrhilabrus katherinae* | Labridae | *Pk* |  | 2 |  |  |  |  |  |  |  |  |  |  |
| *Epibulus insidiator* | Labridae | *MIF* |  |  |  |  |  |  |  |  | 1 |  | 1 | 1 |
| *Gomphosus varius* | Labridae | *MIF* | 3 | 1 | 1 | 1 |  |  | 1 |  |  |  |  |  |
| *Halichoeres hartzfeldii* | Labridae | *MIF* |  |  |  |  |  | 3 |  |  |  |  |  |  |
| *Halichoeres hortulanus* | Labridae | *MIF* | 1 | 4 | 3 | 2 | 1 |  |  |  |  |  |  |  |
| *Halichoeres marginatus* | Labridae | *MIF* |  | 1 |  |  |  |  |  |  |  |  |  |  |
| *Labroides dimidiatus* | Labridae | *MIF* | 2 | 1 | 4 |  | 3 | 2 | 2 |  |  |  |  | 1 |
| *Labroides pectoralis* | Labridae | *MIF* | 1 |  |  |  |  |  |  |  |  | 1 |  |  |
| *Oxycheilinus unifasciatus* | Labridae | *Ps* |  |  |  |  |  | 1 |  |  |  |  |  |  |
| *Thalassoma lutescens* | Labridae | *MIF* | 3 |  | 2 | 2 | 2 | 2 |  |  | 3 |  | 1 |  |
| *Chlorurus sordidus* | Labridae, subfamily Scarinae | *H* |  | 3 |  |  | 1 |  |  |  |  |  |  |  |
| *Scarus niger* | Labridae, subfamily Scarinae | *H* |  |  | 3 | 2 |  | 5 |  |  |  |  |  | 1 |
| *Scarus rubroviolaceus* | Labridae, subfamily Scarinae | *H* |  | 2 | 1 |  |  |  |  |  |  |  |  |  |
| *Scarus spinus* | Labridae, subfamily Scarinae | *H* |  |  |  |  |  |  |  |  |  |  | 1 | 1 |
| *Lutjanus semicinctus* | Lutjanidae | *Ps* |  |  |  |  |  |  |  |  |  |  | 4 |  |
| *Macolor macularis* | Lutjanidae | *Ps* |  |  |  |  |  |  | 2 |  |  |  |  |  |
| *Parupeneus cyclostomus* | Mullidae | *Ps* |  |  |  |  |  |  | 1 |  |  |  |  |  |
| *Parupeneus multifasciatus* | Mullidae | *MIF* |  | 1 | 1 |  | 1 |  | 1 |  |  |  |  |  |
| *Parupeneus trifasciatus* | Mullidae | *MIF* |  |  | 7 |  |  |  |  |  |  |  |  |  |
| *Gymnothorax flavimarginatus* | Muraenidae | *Ps* |  |  |  |  |  |  |  |  | 1 |  |  |  |
| *Centropyge flavissima* | Pomacanthidae | *H* |  |  |  |  |  | 1 |  |  |  |  |  |  |
| *Centropyge loriculus* | Pomacanthidae | *H* |  |  |  |  | 1 |  |  |  |  |  |  |  |
| *Centropyge vroliki* | Pomacanthidae | *H* |  | 2 |  | 1 |  | 2 |  | 1 |  | 1 | 1 |  |
| *Pygoplites diacanthus* | Pomacanthidae | *SIF* | 1 |  | 1 |  |  |  |  |  |  |  |  |  |
| *Amphiprion chrysopterus* | Pomacentridae | *Ps* |  |  | 2 |  | 3 |  |  |  |  |  | 2 |  |
| *Chromis agilis* | Pomacentridae | *Pk* |  |  |  |  | 2 |  |  |  |  |  |  |  |
| *Chromis alpha* | Pomacentridae | *Pk* |  |  | 1 |  |  |  |  |  |  |  |  |  |
| *Chromis margaritifer* | Pomacentridae | *Pk* |  |  | 13 | 4 | 1 | 13 |  | 5 |  | 6 | 7 |  |
| *Chromis ternatensis* | Pomacentridae | *Pk* |  |  |  |  |  |  | 5 |  |  |  |  | 15 |
| *Chrysiptera oxycephala* | Pomacentridae | *Pk* |  | 14 | 2 |  |  |  | 2 |  |  |  |  | 5 |
| *Dascyllus trimaculatus* | Pomacentridae | *Pk* |  |  |  |  | 1 |  |  |  |  |  | 1 |  |
| *Plectroglyphidodon lacrymatus* | Pomacentridae | *H* | 1 | 2 | 6 |  | 5 | 3 |  |  |  |  | 3 |  |
| *Pomacentrus moluccensis* | Pomacentridae | *H* |  |  |  |  |  |  |  |  |  |  | 2 |  |
| *Pomacentrus philippinus* | Pomacentridae | *Pk* |  |  |  |  |  |  | 3 |  |  |  |  |  |
| *Pomacentrus vaiuli* | Pomacentridae | *H* |  |  |  | 1 |  |  |  |  |  |  |  |  |
| *Pictichromis paccagnellae* | Pseudochromidae | *Pk* | 3 |  |  |  |  |  |  |  |  |  |  |  |
| *Cephalopholis argus* | Serranidae | *Ps* |  |  |  |  | 1 |  |  |  | 1 |  |  |  |
| *Epinephelus polyphekadion* | Serranidae | *Ps* |  |  | 1 | 1 |  | 1 |  |  |  |  |  |  |
| *Pseudanthias dispar* | Serranidae, subfamily Anthiadinae | *Pk* |  |  |  |  |  | 35 |  |  |  |  |  |  |
| *Siganus vulpinus* | Siganidae | *H* |  |  |  |  |  |  |  | 1 |  |  |  |  |
| *Arothron nigropunctatus* | Tetraodontidae | *C* |  | 1 |  |  |  |  |  |  |  |  |  |  |
| *Zanclus cornutus* | Zanclidae | *SIF* |  | 1 |  | 1 |  |  |  |  |  |  | 2 |  |

| 30 meters |  |  |  |  |  |  |  |  |  |  |  |  |  |  |
| --- | --- | --- | --- | --- | --- | --- | --- | --- | --- | --- | --- | --- | --- | --- |
|  |  |  | Survey Number | | | | | | | | | | | |
| **Species** | **Family** | **Diet** | **1** | **2** | **3** | **4** | **5** | **6** | **7** | **8** | **9** | **10** | **11** | **12** |
| *Acanthurus nigricans* | Acanthuridae | *Ps* | 1 |  |  |  | 2 |  |  |  |  |  |  |  |
| *Acanthurus thompsoni* | Acanthuridae | *H* |  |  |  | 2 |  |  |  | 1 |  | 2 |  |  |
| *Ctenochaetus binotatus* | Acanthuridae | *D* |  |  |  |  |  |  |  |  |  |  |  |  |
| *Ctenochaetus hawaiiensis* | Acanthuridae | *D* |  |  |  |  |  |  | 4 | 2 |  |  |  | 2 |
| *Ctenochaetus striatus* | Acanthuridae | *D* | 1 | 5 | 2 | 3 | 9 | 14 | 1 |  | 7 |  |  | 7 |
| *Naso lituratus* | Acanthuridae | *Pk* |  |  | 1 |  | 1 |  |  |  |  |  |  |  |
| *Naso unicornis* | Acanthuridae | *Pk* |  |  |  |  |  | 1 |  |  |  |  |  |  |
| *Zebrasoma scopas* | Acanthuridae | *H* | 2 | 5 |  |  |  |  |  |  | 1 |  |  |  |
| *Zebrasoma veliferum* | Acanthuridae | *H* |  |  |  |  | 2 |  |  |  |  |  |  |  |
| *Cheilodipterus macrodon* | Apogonidae | *Pk* |  |  | 1 |  |  |  |  |  |  |  |  |  |
| *Balistapus undulatus* | Balistidae | *MIF* |  |  |  |  |  |  |  |  |  |  |  | 2 |
| *Balistoides viridescens* | Balistidae | *MIF* |  |  | 1 |  |  |  |  |  |  |  |  |  |
| *Melichthys vidua* | Balistidae | *H* |  |  |  |  |  |  |  |  | 3 |  |  |  |
| *Sufflamen bursa* | Balistidae | *MIF* |  |  |  |  |  |  |  | 1 |  |  |  |  |
| *Meiacanthus atrodorsalis* | Blennidae | *Pk* |  |  | 2 | 2 |  | 4 | 1 |  | 1 |  |  | 4 |
| *Triaenodon obesus* | Carcharhinidae | *Ps* |  |  |  |  | 1 |  |  |  |  |  |  |  |
| *Chaetodon auriga* | Chaetodontidae | *C* |  |  |  |  |  |  |  |  |  |  |  |  |
| *Chaetodon kleinii* | Chaetodontidae | *C* |  |  | 1 |  |  |  |  |  |  |  |  |  |
| *Chaetodon mertensii* | Chaetodontidae | *MIF* |  |  |  |  |  |  | 1 | 2 |  |  |  |  |
| *Chaetodon punctatofasciatus* | Chaetodontidae | *C* |  |  |  |  |  |  |  |  |  | 1 |  |  |
| *Chaetodon semeion* | Chaetodontidae | *C* |  |  |  |  | 1 |  |  |  |  |  |  |  |
| *Chaetodon ulietensis* | Chaetodontidae | *C* |  |  |  |  | 1 |  |  |  |  |  |  |  |
| *Forcipiger flavissimus* | Chaetodontidae | *MIF* |  |  |  | 2 |  |  |  | 1 |  |  |  |  |
| *Forcipiger longirostris* | Chaetodontidae | *MIF* | 1 |  |  | 1 | 2 |  |  |  | 1 |  |  |  |
| *Hemitaurichthys polylepis* | Chaetodontidae | *Pk* |  |  |  | 4 |  |  |  |  | 2 |  |  |  |
| *Heniochus chrysostomus* | Chaetodontidae | *Pk* |  |  | 1 |  |  |  |  |  |  |  |  |  |
| *Heniochus singularius* | Chaetodontidae | *C* |  | 1 |  |  |  |  |  |  |  |  |  |  |
| *Myripristis adusta* | Holocentridae | *Pk* |  |  |  |  | 3 |  |  |  |  |  | 1 |  |
| *Myripristis violacea* | Holocentridae | *Pk* |  |  | 2 | 1 |  |  |  |  |  |  |  |  |
| *Bodianus mesothorax* | Labridae | *MIF* |  |  | 2 |  |  |  |  |  |  |  |  |  |
| *Cheilinus fasciatus* | Labridae | *MIF* |  | 16 |  |  |  | 1 | 2 | 1 |  |  |  |  |
| *Halichoeres hortulanus* | Labridae | *MIF* |  | 24 |  |  |  |  |  |  |  |  |  |  |
| *Halichoeres marginatus* | Labridae | MIF |  |  |  |  |  |  |  |  |  |  |  | 1 |
| *Halichoeres melasmapomus* | Labridae | MIF |  |  |  |  |  | 1 |  |  |  |  |  |  |
| *Labrichthys unilineatus* | Labridae | C |  |  |  |  |  |  | 1 |  |  |  |  |  |
| *Labroides dimidiatus* | Labridae | *MIF* | 1 |  | 1 | 1 |  | 1 |  |  |  |  |  | 3 |
| *Oxycheilinus orientalis* | Labridae | *Ps* | 1 |  |  |  |  |  |  |  | 1 |  |  | 2 |
| *Pseudocheilinus evanidus* | Labridae | *MIF* |  |  |  |  | 1 | 1 |  |  |  |  |  |  |
| *Pseudocheilinus octotaenia* | Labridae | *MIF* |  | 1 |  |  |  |  |  |  |  |  |  |  |
| *Thalassoma lutescens* | Labridae | *MIF* |  |  |  |  |  | 1 |  |  |  |  |  |  |
| *Wetmorella nigropinnata* | Labridae | *MIF* |  | 1 |  |  | 1 |  |  | 2 |  |  |  |  |
| *Scarus niger* | Labridae, subfamily Scarinae | *H* |  |  |  |  | 1 |  |  |  |  |  | 1 |  |
| *Scarus rubroviolaceus* | Labridae, subfamily Scarinae | *H* |  |  |  | 2 | 2 |  |  | 2 |  |  |  |  |
| *Scarus spinus* | Labridae, subfamily Scarinae | *H* |  |  |  |  | 1 |  |  |  |  |  |  |  |
| *Gnathodentex aureolineatus* | Lethrinidae | *MIF* |  |  |  |  | 1 |  |  |  |  |  |  |  |
| *Lutjanus bohar* | Lutjanidae | *Ps* |  |  |  |  | 1 |  |  |  |  |  | 1 |  |
| *Lutjanus decussatus* | Lutjanidae | *MIF* | 1 | 2 |  |  |  |  |  |  |  |  |  |  |
| *Lutjanus semicinctus* | Lutjanidae | *Ps* |  |  |  |  |  |  |  |  |  |  | 1 |  |
| *Caesio caerulaurea* | Lutjanidae, subfamily Caesioninae | *Ps* |  | 1 |  |  |  |  |  |  |  |  |  |  |
| *Pterocaesio tile* | Lutjanidae, subfamily Caesioninae | Pk |  |  |  |  |  |  |  |  |  |  |  | 1 |
| *Parupeneus multifasciatus* | Mullidae | *MIF* |  | 1 |  |  |  |  | 1 | 1 | 1 |  |  |  |
| *Gymnothorax flavimarginatus* | Muraenidae | *Ps* |  |  |  |  | 1 |  |  |  |  |  |  |  |
| *Centropyge bispinosa* | Pomacanthidae | *H* |  |  |  |  | 2 |  |  |  |  |  |  |  |
| *Centropyge flavissima* | Pomacanthidae | *H* |  |  |  |  |  | 1 |  |  |  |  |  |  |
| *Centropyge heraldi* | Pomacanthidae | *H* |  |  |  |  |  | 1 |  |  |  |  |  |  |
| *Centropyge multicolor* | Pomacanthidae | *H* |  |  |  |  |  |  |  | 1 |  |  | 1 |  |
| *Centropyge vroliki* | Pomacanthidae | *H* |  |  |  |  | 1 |  |  |  |  |  |  |  |
| *Pygoplites diacanthus* | Pomacanthidae | *SIF* |  |  |  |  |  |  |  |  | 1 |  |  |  |
| *Amblyglyphidodon aureus* | Pomacentridae | *Pk* | 5 |  | 5 |  |  |  |  | 1 | 2 |  |  |  |
| *Chromis acares* | Pomacentridae | Pk |  |  |  |  | 3 |  |  |  |  |  |  |  |
| *Chromis agilis* | Pomacentridae | Pk |  |  |  | 1 |  |  |  |  |  |  |  |  |
| *Chromis alpha* | Pomacentridae | Pk |  | 1 |  |  | 1 |  | 2 | 5 | 2 | 2 | 1 |  |
| *Chromis amboinensis* | Pomacentridae | Pk |  |  |  | 2 |  |  |  |  |  |  |  |  |
| *Chromis margaritifer* | Pomacentridae | Pk |  |  |  |  |  | 2 |  |  |  |  |  |  |
| *Chromis ternatensis* | Pomacentridae | Pk |  | 5 | 4 |  |  |  | 11 | 22 | 19 | 4 | 9 | 3 |
| *Plectroglyphidodon lacrymatus* | Pomacentridae | *H* |  |  |  |  |  |  |  |  |  |  |  |  |
| *Pomacentrus vaiuli* | Pomacentridae | *H* |  |  |  |  |  | 1 |  |  |  |  |  |  |
| *Acanthochromis polyacanthus* | Serranidae | *Ps* |  |  | 3 |  |  |  |  |  |  |  |  |  |
| *Cephalopholis argus* | Serranidae | *Ps* |  |  | 1 |  |  |  |  |  |  |  |  |  |
| *Cephalopholis spiloparaea* | Serranidae | *Ps* |  |  |  |  |  |  |  |  |  |  | 2 | 1 |
| *Epinephelus polyphekadion* | Serranidae | *Ps* |  |  | 1 |  |  |  |  |  |  |  |  |  |
| *Pseudanthias pascalus* | Serranidae, subfamily Anthiadinae | *Pk* |  |  |  | 17 |  |  |  |  | 3 |  |  |  |
| *Pseudanthias smithvanizi* | Serranidae, subfamily Anthiadinae | *Pk* |  |  |  |  |  |  |  |  | 2 |  |  |  |
| *Zanclus cornutus* | Zanclidae | *SIF* |  |  | 4 |  | 2 |  |  |  |  |  |  |  |

| 40 meters |  |  |  |  |  |  |  |  |  |  |  |  |  |  |
| --- | --- | --- | --- | --- | --- | --- | --- | --- | --- | --- | --- | --- | --- | --- |
|  |  |  | Survey Number | | | | | | | | | | | |
| **Species** | **Family** | **Diet** | **1** | **2** | **3** | **4** | **5** | **6** | **7** | **8** | **9** | **10** | **11** | **12** |
| *Acanthurus nigricans* | Acanthuridae | *H* |  |  |  |  | 2 |  |  |  |  |  |  |  |
| *Acanthurus pyroferus* | Acanthuridae | *H* |  |  |  |  |  |  |  | 1 |  |  |  |  |
| *Acanthurus thompsoni* | Acanthuridae | *H* |  |  |  | 2 |  |  |  | 2 |  |  | 1 |  |
| *Ctenochaetus hawaiiensis* | Acanthuridae | *D* |  |  |  |  |  | 1 |  |  |  |  |  |  |
| *Ctenochaetus striatus* | Acanthuridae | *D* |  |  | 5 |  | 12 | 7 |  | 1 | 1 | 1 |  |  |
| *Naso unicornis* | Acanthuridae | *Pk* |  |  |  |  |  |  |  | 2 |  |  |  |  |
| *Zebrasoma scopas* | Acanthuridae | *H* |  | 3 |  |  |  | 3 |  |  |  |  |  |  |
| *Zebrasoma veliferum* | Acanthuridae | *H* |  |  |  |  |  |  | 2 | 2 |  |  |  |  |
| *Balistapus undulatus* | Balistidae | *MIF* |  |  |  |  | 1 | 1 |  |  | 3 |  |  |  |
| *Sufflamen bursa* | Balistidae | *MIF* |  | 1 |  |  |  |  |  | 1 |  | 1 |  |  |
| *Meiacanthus atrodorsalis* | Blennidae | *Pk* |  |  |  |  | 1 |  |  |  |  |  |  |  |
| *Caranx melampygus* | Carangidae | *Ps* |  |  |  |  |  |  |  |  |  |  |  | 2 |
| *Chaetodon bennetti* | Chaetodontidae | *C* |  |  |  |  |  |  |  |  |  |  | 1 |  |
| *Chaetodon lineolatus* | Chaetodontidae | *C* |  |  |  | 1 |  |  |  |  |  |  |  |  |
| *Chaetodon mertensii* | Chaetodontidae | *MIF* |  |  |  |  |  |  |  | 1 |  |  |  |  |
| *Chaetodon punctatofasciatus* | Chaetodontidae | *C* |  |  |  |  |  | 2 |  |  |  |  |  |  |
| *Chaetodon ulietensis* | Chaetodontidae | *C* |  |  |  | 2 |  |  |  |  |  |  |  |  |
| *Forcipiger flavissimus* | Chaetodontidae | *MIF* |  |  |  |  | 1 |  |  | 4 |  |  |  |  |
| *Forcipiger longirostris* | Chaetodontidae | *MIF* |  | 1 |  |  |  |  | 2 | 2 |  |  |  |  |
| *Hemitaurichthys polylepis* | Chaetodontidae | *Pk* |  |  |  |  |  |  |  |  |  |  |  | 3 |
| *Myripristis kuntee* | Holocentridae | *Pk* | 1 |  |  |  |  | 1 | 2 |  |  |  |  |  |
| *Bodianus mesothorax* | Labridae | *MIF* |  |  | 1 |  |  |  |  |  |  |  |  |  |
| *Cheilinus fasciatus* | Labridae | *MIF* |  | 1 | 1 |  | 1 |  |  |  |  |  |  |  |
| *Epibulus insidiator* | Labridae | *MIF* |  |  |  |  | 1 |  |  |  |  |  |  |  |
| *Halichoeres hortulanus* | Labridae | *MIF* |  |  |  |  |  | 1 |  |  |  |  |  |  |
| *Halichoeres melasmapomus* | Labridae | *MIF* |  |  |  |  |  |  | 1 |  |  |  |  |  |
| *Labroides dimidiatus* | Labridae | *MIF* |  | 1 |  |  |  |  | 1 |  |  | 1 |  |  |
| *Oxycheilinus orientalis* | Labridae | *Ps* |  | 1 | 1 |  |  |  |  |  |  |  |  |  |
| *Pseudocheilinus evanidus* | Labridae | *MIF* |  |  |  |  |  |  | 1 |  |  | 1 |  |  |
| *Pseudocheilinus octotaenia* | Labridae | *MIF* |  | 2 |  |  |  |  |  |  |  |  |  |  |
| *Chlorurus sordidus* | Labridae, subfamily Scarinae | *H* |  |  |  |  |  |  | 4 |  |  |  |  |  |
| *Scarus fuscocaudalis* | Labridae, subfamily Scarinae | *H* |  |  |  |  | 1 |  |  |  |  |  |  |  |
| *Scarus niger* | Labridae, subfamily Scarinae | *H* |  |  |  |  |  |  |  |  |  |  |  | 1 |
| *Lutjanus decussatus* | Lutjanidae | *MIF* | 6 |  |  |  |  |  |  |  |  |  |  |  |
| *Caesio caerulaurea* | Lutjanidae, subfamily Caesioninae | *Ps* |  |  |  |  |  | 4 |  |  |  |  |  |  |
| *Parupeneus crassilabris* | Mullidae | *MIF* |  |  |  |  |  |  |  |  |  |  | 1 |  |
| *Parupeneus multifasciatus* | Mullidae | *MIF* |  | 1 | 1 |  |  | 1 |  |  |  |  |  | 1 |
| *Centropyge bispinosa* | Pomacanthidae | *H* |  |  |  |  | 1 |  |  |  |  | 1 |  |  |
| *Centropyge heraldi* | Pomacanthidae | *H* |  |  |  |  | 1 |  |  |  |  |  |  |  |
| *Centropyge multicolor* | Pomacanthidae | *H* |  |  |  |  | 1 |  | 2 |  |  |  |  |  |
| *Pygoplites diacanthus* | Pomacanthidae | *SIF* |  |  |  |  |  |  | 1 |  |  | 1 |  | 1 |
| *Amblyglyphidodon aureus* | Pomacentridae | *Pk* |  |  | 3 |  |  |  | 4 | 1 |  |  |  | 1 |
| *Amphiprion chrysopterus* | Pomacentridae | *Ps* |  |  |  |  |  |  |  |  |  |  | 2 |  |
| *Chromis alpha* | Pomacentridae | *Pk* |  | 7 | 6 |  |  | 12 | 2 | 1 | 11 | 3 | 2 |  |
| *Chromis delta* | Pomacentridae | *Pk* |  |  |  |  |  |  |  |  |  |  | 2 | 6 |
| *Chromis margaritifer* | Pomacentridae | *Pk* |  | 2 |  |  |  |  |  |  |  |  |  |  |
| *Chromis ternatensis* | Pomacentridae | *Pk* |  | 6 | 29 | 6 | 6 |  | 24 | 1 | 9 | 4 | 13 | 11 |
| *Chromis xanthura* | Pomacentridae | *Pk* |  |  |  |  |  | 1 |  |  |  |  |  |  |
| *Chrysiptera caeruleolineata* | Pomacentridae | *Pk* |  | 3 | 1 |  |  |  |  | 1 |  |  |  |  |
| *Cephalopholis spiloparaea* | Serranidae | *Ps* |  |  |  | 2 |  | 1 | 1 |  |  | 1 | 2 | 2 |
| *Pseudanthias pascalus* | Serranidae, subfamily Anthiadinae | *Pk* |  |  |  |  |  |  | 21 |  |  |  | 4 | 8 |
| *Pseudanthias smithvanizi* | Serranidae, subfamily Anthiadinae | *Pk* |  |  |  |  |  |  |  |  |  |  |  | 1 |

| 50 meters |  |  |  |  |  |  |  |  |  |  |  |  |  |  | |
| --- | --- | --- | --- | --- | --- | --- | --- | --- | --- | --- | --- | --- | --- | --- | --- |
|  |  |  | Survey Number | | | | | | | | | | | |  |
| **Species** | **Family** | **Diet** | **1** | **2** | **3** | **4** | **5** | **6** | **7** | **8** | **9** | **10** | **11** | **12** | |
| *Acanthurus pyroferus* | Acanthuridae | *H* |  | 1 |  |  |  |  |  |  |  |  |  |  | |
| *Acanthurus thompsoni* | Acanthuridae | *H* | 2 |  |  |  |  |  | 2 |  | 2 | 7 |  |  | |
| *Ctenochaetus striatus* | Acanthuridae | *D* | 1 | 2 | 2 |  |  |  | 2 |  |  | 9 | 4 |  | |
| *Naso lituratus* | Acanthuridae | *Pk* | 2 |  |  |  |  |  |  |  |  |  |  |  | |
| *Naso unicornis* | Acanthuridae | *Pk* |  |  |  |  |  | 2 |  |  |  |  |  |  | |
| *Zebrasoma scopas* | Acanthuridae | *H* |  |  |  |  |  |  |  |  |  | 2 |  |  | |
| *Cheilodipterus isostigmus* | Apogonidae | *Pk* |  |  |  |  |  |  |  | 2 |  |  |  |  | |
| *Balistapus undulatus* | Balistidae | *MIF* | 1 |  |  | 2 |  |  |  |  | 1 | 1 |  |  | |
| *Balistoides viridescens* | Balistidae | *MIF* |  |  |  |  |  |  |  |  | 1 |  |  |  | |
| *Sufflamen bursa* | Balistidae | *MIF* |  |  |  |  |  |  |  |  |  | 1 |  | 1 | |
| *Meiacanthus atrodorsalis* | Blennidae | *Pk* |  | 1 |  |  |  |  |  |  |  |  |  |  | |
| *Caranx melampygus* | Carangidae | *Ps* |  |  |  |  |  |  | 1 |  |  |  |  |  | |
| *Triaenodon obesus* | Carcharhinidae | *Ps* |  | 1 |  |  |  |  |  |  |  |  |  |  | |
| *Chaetodon auriga* | Chaetodontidae | *C* |  | 1 |  |  |  |  |  |  |  |  |  |  | |
| *Chaetodon bennetti* | Chaetodontidae | *C* |  |  |  |  |  |  |  |  |  | 1 |  |  | |
| *Chaetodon lunulatus* | Chaetodontidae | *C* | 2 |  | 1 |  |  |  |  |  |  |  |  |  | |
| *Chaetodon mertensii* | Chaetodontidae | *MIF* |  |  | 2 |  |  |  |  |  |  |  |  |  | |
| *Chaetodon punctatofasciatus* | Chaetodontidae | *C* |  |  |  |  | 3 | 1 |  | 1 | 2 |  | 1 |  | |
| *Forcipiger flavissimus* | Chaetodontidae | *MIF* |  | 2 |  |  |  |  | 3 | 5 | 2 |  |  |  | |
| *Forcipiger longirostris* | Chaetodontidae | *MIF* |  |  | 2 |  |  | 3 |  | 4 | 1 |  |  | 1 | |
| *Nemateleotris helfrichi* | Gobiidae | *Pk* |  |  |  |  |  |  |  |  | 1 |  |  |  | |
| *Myripristis kuntee* | Holocentridae | *Pk* |  |  |  |  | 6 |  |  | 7 |  | 4 |  |  | |
| *Bodianus mesothorax* | Labridae | *MIF* |  |  |  |  |  |  | 1 |  |  | 1 | 1 |  | |
| *Cheilinus fasciatus* | Labridae | *MIF* | 2 |  |  | 1 |  |  |  | 1 |  | 2 | 1 |  | |
| *Cirrhilabrus katherinae* | Labridae | *Pk* |  |  | 1 |  |  |  |  |  |  |  |  |  | |
| *Epibulus insidiator* | Labridae | *MIF* |  |  |  |  |  |  | 1 |  |  |  |  |  | |
| *Halichoeres melasmapomus* | Labridae | *MIF* |  |  |  |  | 1 |  |  |  |  |  |  |  | |
| *Halichoeres melasmapomus* | Labridae | *MIF* |  |  |  |  |  |  |  | 2 |  |  |  | 1 | |
| *Labroides dimidiatus* | Labridae | *MIF* |  |  |  |  |  |  | 1 |  |  |  |  | 1 | |
| *Macropharyngodon meleagris* | Labridae | *SIF* |  |  |  | 1 |  |  |  |  |  |  |  |  | |
| *Oxycheilinus arenatus* | Labridae | *Ps* |  |  |  |  | 1 |  |  |  |  |  |  |  | |
| *Pseudocheilinus evanidus* | Labridae | *MIF* |  |  | 1 |  |  |  |  |  |  |  |  |  | |
| *Chlorurus sordidus* | Labridae, subfamily Scarinae | *H* |  |  | 1 |  |  |  |  |  | 1 |  |  |  | |
| *Scarus fuscocaudalis* | Labridae, subfamily Scarinae | *H* |  |  | 2 |  |  |  |  |  |  |  |  |  | |
| *Monotaxis grandoculis* | Lethrinidae | *MIF* |  | 1 |  |  |  |  |  |  |  | 1 |  |  | |
| *Lutjanus bohar* | Lutjanidae | *Ps* |  | 1 |  |  |  | 1 |  |  |  |  |  |  | |
| *Lutjanus decussatus* | Lutjanidae | *MIF* |  |  | 1 |  |  |  |  |  |  |  |  |  | |
| *Lutjanus semicinctus* | Lutjanidae | *Ps* |  |  |  |  |  |  |  | 1 |  |  |  |  | |
| *Macolor macularis* | Lutjanidae | *Ps* |  |  |  |  |  |  |  |  |  |  | 1 |  | |
| *Caesio caerulaurea* | Lutjanidae, subfamily Caesioninae | *Ps* |  | 3 |  |  |  |  |  |  |  |  |  | 6 | |
| *Pterocaesio pisang* | Lutjanidae, subfamily Caesioninae | *Pk* | 3 |  |  | 3 |  |  |  |  |  |  |  |  | |
| *Parupeneus multifasciatus* | Mullidae | *MIF* |  |  |  |  |  |  | 2 |  | 2 |  |  | 1 | |
| *Centropyge heraldi* | Pomacanthidae | *H* | 2 | 2 |  |  |  |  |  |  |  |  |  |  | |
| *Centropyge multicolor* | Pomacanthidae | *H* | 4 |  |  | 1 |  |  |  |  | 1 |  |  | 1 | |
| *Pygoplites diacanthus* | Pomacanthidae | *SIF* |  |  |  |  |  |  | 3 |  | 1 |  |  | 1 | |
| *Amblyglyphidodon aureus* | Pomacentridae | *Pk* |  |  |  |  |  | 3 |  |  |  |  | 1 |  | |
| *Chromis acares* | Pomacentridae | *Pk* | 16 | 7 |  |  |  |  |  |  |  |  |  |  | |
| *Chromis alpha* | Pomacentridae | *Pk* |  |  | 5 | 11 | 3 |  | 17 | 1 | 11 |  |  | 1 | |
| *Chromis delta* | Pomacentridae | *Pk* |  |  |  |  |  |  | 3 | 1 |  |  |  |  | |
| *Chromis ternatensis* | Pomacentridae | *Pk* |  |  |  |  | 21 | 14 | 3 | 28 | 8 | 15 | 17 | 17 | |
| *Priacanthus hamrur* | Priacanthidae | *Pk* |  |  |  |  |  |  |  |  |  | 1 |  |  | |
| *Gymnosarda unicolor* | Scombridae | *Ps* |  |  |  |  | 4 |  | 1 |  |  |  |  |  | |
| *Pterois antennata* | Scorpaenidae | *MIF* |  |  |  |  |  |  |  |  |  |  | 1 |  | |
| *Cephalopholis spiloparaea* | Serranidae | *Ps* | 2 |  |  | 2 |  | 2 |  | 1 |  | 2 |  | 1 | |
| *Epinephelus polyphekadion* | Serranidae | *Ps* |  |  |  |  | 9 |  |  |  |  |  |  |  | |
| *Pseudanthias cooperi* | Serranidae, subfamily Anthiadinae | *Pk* |  |  |  |  | 15 |  |  | 25 |  |  |  |  | |
| *Pseudanthias pascalus* | Serranidae, subfamily Anthiadinae | *Pk* |  |  |  |  | 5 |  |  | 7 |  |  |  |  | |
| *Pseudanthias pleurotaenia* | Serranidae, subfamily Anthiadinae | *Pk* |  |  |  |  | 5 | 1 |  |  |  |  |  |  | |
| *Pseudanthias randalli* | Serranidae, subfamily Anthiadinae | *Pk* |  |  |  |  | 3 |  |  |  |  |  |  |  | |
| *Pseudanthias smithvanizi* | Serranidae, subfamily Anthiadinae | *Pk* |  |  |  |  | 4 |  |  |  |  |  |  |  | |
| *Zanclus cornutus* | Zanclidae | *SIF* |  |  |  |  |  |  |  |  |  | 1 |  |  | |

| 60 meters |  |  |  |  |  |  |  |  |  |  |  |  |  |  | |
| --- | --- | --- | --- | --- | --- | --- | --- | --- | --- | --- | --- | --- | --- | --- | --- |
|  |  |  | Survey Number | | | | | | | | | | | |  |
| **Species** | **Family** | **Diet** | **1** | **2** | **3** | **4** | **5** | **6** | **7** | **8** | **9** | **10** | **11** | **12** | |
| *Acanthurus pyroferus* | Acanthuridae | *H* |  | 1 |  |  |  |  |  |  |  |  |  |  | |
| *Acanthurus thompsoni* | Acanthuridae | *H* | 2 |  |  |  |  |  | 2 |  | 2 | 7 |  |  | |
| *Ctenochaetus striatus* | Acanthuridae | *D* | 1 | 2 | 2 |  |  |  | 2 |  |  | 9 | 4 |  | |
| *Naso lituratus* | Acanthuridae | *Pk* | 2 |  |  |  |  |  |  |  |  |  |  |  | |
| *Naso unicornis* | Acanthuridae | *Pk* |  |  |  |  |  | 2 |  |  |  |  |  |  | |
| *Zebrasoma scopas* | Acanthuridae | *H* |  |  |  |  |  |  |  |  |  | 2 |  |  | |
| *Cheilodipterus isostigmus* | Apogonidae | *Pk* |  |  |  |  |  |  |  | 2 |  |  |  |  | |
| *Balistapus undulatus* | Balistidae | *MIF* | 1 |  |  | 2 |  |  |  |  | 1 | 1 |  |  | |
| *Balistoides viridescens* | Balistidae | *MIF* |  |  |  |  |  |  |  |  | *1* |  |  |  | |
| *Sufflamen bursa* | Balistidae | *MIF* |  |  |  |  |  |  |  |  |  | 1 |  | 1 | |
| *Meiacanthus atrodorsalis* | Blennidae | *Pk* |  | 1 |  |  |  |  |  |  |  |  |  |  | |
| *Caranx melampygus* | Carangidae | *Ps* |  |  |  |  |  |  | 1 |  |  |  |  |  | |
| *Triaenodon obesus* | Carcharhinidae | *Ps* |  | 1 |  |  |  |  |  |  |  |  |  |  | |
| *Chaetodon auriga* | Chaetodontidae | *C* |  | 1 |  |  |  |  |  |  |  |  |  |  | |
| *Chaetodon lunulatus* | Chaetodontidae | *C* | 2 |  | 1 |  |  |  |  |  |  |  |  |  | |
| *Chaetodon mertensii* | Chaetodontidae | *MIF* |  |  | 2 |  |  |  |  |  |  |  |  |  | |
| *Chaetodon punctatofasciatus* | Chaetodontidae | *C* |  |  |  |  | 3 | 1 |  | 1 | 2 |  | 1 |  | |
| *Forcipiger flavissimus* | Chaetodontidae | *MIF* |  | 2 |  |  |  |  | 3 | 5 | 2 |  |  |  | |
| *Forcipiger longirostris* | Chaetodontidae | *MIF* |  | 1 |  |  |  |  | 3 | 4 | 1 |  |  |  | |
| *Nemateleotris helfrichi* | Gobiidae | *Pk* |  |  |  |  |  |  |  |  | 1 |  |  |  | |
| *Myripristis kuntee* | Holocentridae | *Pk* |  |  |  |  | 6 |  |  | 7 |  | 4 |  |  | |
| *Bodianus mesothorax* | Labridae | *MIF* |  |  |  |  |  |  | 1 |  |  | 2 |  |  | |
| *Cheilinus fasciatus* | Labridae | *MIF* | 2 |  |  | 1 |  |  |  | 1 |  | 2 | 1 |  | |
| *Cirrhilabrus katherinae* | Labridae | *Pk* |  |  |  |  | 1 |  |  |  |  |  |  |  | |
| *Epibulus insidiator* | Labridae | *MIF* |  |  |  |  |  |  | 1 |  |  |  |  |  | |
| *Halichoeres hartzfeldii* | Labridae | *MIF* |  |  | 1 |  |  |  |  |  |  |  |  |  | |
| *Halichoeres melasmapomus* | Labridae | *MIF* |  |  |  |  | 1 |  |  |  |  |  |  |  | |
| *Halichoeres melasmapomus* | Labridae | *MIF* |  |  |  |  |  |  |  | 2 |  |  |  | 1 | |
| *Labroides dimidiatus* | Labridae | *MIF* |  |  |  |  |  |  | 1 |  |  |  |  | 1 | |
| *Macropharyngodon meleagris* | Labridae | *SIF* |  |  |  | 1 |  |  |  |  |  |  |  |  | |
| *Pseudocheilinus evanidus* | Labridae | *MIF* |  |  |  |  |  |  |  |  |  |  |  | 2 | |
| *Pseudocheilinus evanidus* | Labridae | *MIF* |  |  | 1 |  |  |  |  |  |  |  |  |  | |
| *Chlorurus sordidus* | Labridae, subfamily Scarinae | *H* |  |  | 1 |  |  |  |  |  | 1 |  |  |  | |
| *Scarus fuscocaudalis* | Labridae, subfamily Scarinae | *H* |  |  | 2 |  |  |  |  |  |  |  |  |  | |
| *Monotaxis grandoculis* | Lethrinidae | *MIF* |  | 1 |  |  |  |  |  |  |  | 1 |  |  | |
| *Lutjanus bohar* | Lutjanidae | *Ps* |  | 1 |  |  |  | 1 |  |  |  |  |  |  | |
| *Lutjanus decussatus* | Lutjanidae | *MIF* |  |  | 1 |  |  |  |  |  |  |  |  |  | |
| *Lutjanus semicinctus* | Lutjanidae | *Ps* |  |  |  |  |  |  |  | 1 |  |  |  |  | |
| *Macolor macularis* | Lutjanidae | *Ps* |  |  |  |  |  |  |  |  |  |  | 1 |  | |
| *Caesio caerulaurea* | Lutjanidae, subfamily Caesioninae | *Ps* |  | 30 |  |  |  |  |  |  |  |  |  | 6 | |
| *Pterocaesio pisang* | Lutjanidae, subfamily Caesioninae | *Pk* | 30 |  |  | 30 |  |  |  |  |  |  |  |  | |
| *Parupeneus multifasciatus* | Mullidae | *MIF* |  |  |  |  |  |  | 2 |  | 2 |  |  | 1 | |
| *Centropyge heraldi* | Pomacanthidae | *H* | 2 | 2 |  |  |  |  |  |  |  |  |  |  | |
| *Centropyge multicolor* | Pomacanthidae | *H* | 4 |  |  | 1 |  |  |  |  | 1 |  |  | 1 | |
| *Pygoplites diacanthus* | Pomacanthidae | *SIF* |  |  |  |  |  |  | 3 |  | 1 |  |  | 1 | |
| *Amblyglyphidodon aureus* | Pomacentridae | *Pk* |  |  |  |  |  | 3 |  |  |  |  | 1 |  | |
| *Chromis acares* | Pomacentridae | *Pk* | 16 | 7 |  |  |  |  |  |  |  |  |  |  | |
| *Chromis alpha* | Pomacentridae | *Pk* |  |  | 5 | 11 | 3 |  | 17 | 1 | 11 |  |  | 10 | |
| *Chromis delta* | Pomacentridae | *Pk* |  |  |  |  |  |  | 3 | 1 |  |  |  |  | |
| *Chromis ternatensis* | Pomacentridae | *Pk* |  |  |  |  | 21 | 14 | 30 | 28 | 8 | 15 | 17 | 17 | |
| *Priacanthus hamrur* | Priacanthidae | *Pk* |  |  |  |  |  |  |  |  |  | 1 |  |  | |
| *Gymnosarda unicolor* | Scombridae | *Ps* |  |  |  |  | 40 |  | 1 |  |  |  |  |  | |
| *Pterois antennata* | Scorpaenidae | *MIF* |  |  |  |  |  |  |  |  |  |  | 1 |  | |
| *Cephalopholis spiloparaea* | Serranidae | *Ps* | 2 |  |  | 2 |  | 2 |  | 1 |  | 2 |  | 1 | |
| *Epinephelus polyphekadion* | Serranidae | *Ps* |  |  |  |  | 9 |  |  |  |  |  |  |  | |
| *Pseudanthias cooperi* | Serranidae, subfamily Anthiadinae | *Pk* |  |  |  |  | 15 |  |  | 25 |  |  |  |  | |
| *Pseudanthias pascalus* | Serranidae, subfamily Anthiadinae | *Pk* |  |  |  |  | 50 |  |  | 7 |  |  |  |  | |
| *Pseudanthias pleurotaenia* | Serranidae, subfamily Anthiadinae | *Pk* |  |  |  |  | 50 | 1 |  |  |  |  |  |  | |
| *Pseudanthias randalli* | Serranidae, subfamily Anthiadinae | *Pk* |  |  |  |  | 3 |  |  |  |  |  |  |  | |
| *Pseudanthias smithvanizi* | Serranidae, subfamily Anthiadinae | *Pk* |  |  |  |  | 40 |  |  |  |  |  |  |  | |
| *Zanclus cornutus* | Zanclidae | *SIF* |  |  |  |  |  |  |  |  |  | 1 |  |  | |
