# Supplementary material for: Shifting reef fish assemblages along a depth gradient in Pohnpei, Micronesia
Source: PeerJ. 2018 Apr 24;6:e4650. doi: 10.7717/peerj.4650 (PMC5922234; doi:10.7717/peerj.4650)
Supplement: Table S4 — Summary of observed (Sobs), Abundance Coverage based Estimator (ACE), and Chao1 species richness estimators for fish assemblages at each depth. [file peerj-06-4650-s004.docx]

**Table S4**. Summary of observed (*S*_obs_), Abundance Coverage based Estimator (ACE), and Chao1 species richness estimators for fish assemblages at each depth.

| Depth (m) | *S*_obs_ | ACE | Chao1 |
| --- | --- | --- | --- |
| 10 | 68 | 78 ± 3 | 77 ± 6 |
| 20 | 69 | 79 ± 3 | 78 ± 6 |
| 30 | 73 | 101 ± 7 | 107 ± 18 |
| 40 | 52 | 65 ± 4 | 64 ± 8 |
| 50 | 61 | 82 ± 0 | 79 ± 11 |
| 60 | 61 | 83 ± 0 | 76 ± 9 |
