# Supplementary material for: Shifting reef fish assemblages along a depth gradient in Pohnpei, Micronesia
Source: PeerJ. 2018 Apr 24;6:e4650. doi: 10.7717/peerj.4650 (PMC5922234; doi:10.7717/peerj.4650)
Supplement: Table S5 — Similarity percentage (SIMPER) analysis based on (a) species, (b) family and (c) trophic position indicating the variable that is contributing the highest percentage at each depth (10–60 m). Contributions up to 75% are reported. Abbreviations: Av.Abund, average abundance; Av.Sim, average similarity; Sim/SD, similarity standard deviation; Contrib%, percent contribution; Cum.%, cumulative contribution. [file peerj-06-4650-s005.docx]

Table S5. Similarity percentage (SIMPER) analysis based on a) species, b) family and c) trophic position indicating the variable that is contributing the highest percentage at each depth (10-60 m). Contributions up to 75% are reported. Abbreviations: Av.Abund, average abundance; Av.Sim, average similiary; Sim/SD, similarity standard deviation; Contrib%, percent contribution; Cum.%, cummularive contribution.

a)

| *10 m* |  |  |  |  |  |
| --- | --- | --- | --- | --- | --- |
| Average similarity: 31.73 |  |  |  |  |  |
|  |  |  |  |  |  |
| Species | Av.Abund | Av.Sim | Sim/SD | Contrib% | Cum.% |
| *Ctenochaetus striatus* | 3.14 | 11.29 | 3.33 | 35.57 | 35.57 |
| *Acanthurus nigricans* | 1.63 | 4.70 | 1.12 | 14.80 | 50.37 |
| *Chromis margaritifer* | 1.46 | 2.45 | 0.61 | 7.71 | 58.08 |
| *Myripristes kuntee* | 0.90 | 1.64 | 0.50 | 5.16 | 63.24 |
| *Thalassoma lutescens* | 0.84 | 1.59 | 0.63 | 5.01 | 68.25 |
| *Labroides dimidiatus* | 0.83 | 1.44 | 0.66 | 4.54 | 72.78 |
| *Plectroglyphidodon lacrymatus* | 0.88 | 1.05 | 0.53 | 3.31 | 76.1 |
| *20 m* |  |  |  |  |  |
| Average similarity: 26.13 |  |  |  |  |  |
|  |  |  |  |  |  |
| Species | Av.Abund | Av.Sim | Sim/SD | Contrib% | Cum.% |
| *Ctenochaetus striatus* | 2.39 | 10.76 | 1.83 | 41.18 | 41.18 |
| *Myripristes kuntee* | 1.13 | 3.00 | 0.71 | 11.47 | 52.64 |
| *Zebrasoma scopas* | 1.07 | 2.47 | 0.65 | 9.46 | 62.1 |
| *Acanthurus nigricans* | 0.63 | 1.61 | 0.52 | 6.15 | 68.25 |
| *Chromis ternatensis* | 1.17 | 1.53 | 0.40 | 5.86 | 74.1 |
| *Chelinius fasciatus* | 0.60 | 1.29 | 0.53 | 4.94 | 79.05 |
| *30 m* |  |  |  |  |  |
| Average similarity: 18.20 |  |  |  |  |  |
|  |  |  |  |  |  |
| Species | Av.Abund | Av.Sim | Sim/SD | Contrib% | Cum.% |
| *Chromis ternatensis* | 2.06 | 7.02 | 0.92 | 38.58 | 38.58 |
| *Ctenochaetus striatus* | 1.43 | 3.18 | 0.74 | 17.45 | 56.03 |
| *Chromis alpha* | 0.79 | 2.18 | 0.59 | 11.99 | 68.02 |
| *Meiacanthus atrodorsalis* | 0.74 | 1.50 | 0.50 | 8.27 | 76.28 |
| *40 m* |  |  |  |  |  |
| Average similarity: 25.89 |  |  |  |  |  |
|  |  |  |  |  |  |
| Species | Av.Abund | Av.Sim | Sim/SD | Contrib% | Cum.% |
| *Chromis ternatensis* | 3.17 | 11.95 | 1.35 | 46.14 | 46.14 |
| *Chromis alpha* | 1.72 | 5.84 | 0.88 | 22.57 | 68.71 |
| *Ctenochaetus striatus* | 0.95 | 1.95 | 0.50 | 7.52 | 76.23 |
| *50 m* |  |  |  |  |  |
| Average similarity: 20.91 |  |  |  |  |  |
|  |  |  |  |  |  |
| Species | Av.Abund | Av.Sim | Sim/SD | Contrib% | Cum.% |
| *Chromis ternatensis* | 2.84 | 7.60 | 0.79 | 36.33 | 36.33 |
| *Chromis alpha* | 1.57 | 3.28 | 0.55 | 15.68 | 52.01 |
| *Ctenochaetus striatus* | 0.85 | 1.67 | 0.51 | 8.00 | 60 |
| *Cephalopholis spiloparaea* | 0.64 | 1.45 | 0.51 | 6.93 | 66.93 |
| *Chelinius fasciatus* | 0.49 | 0.83 | 0.41 | 3.99 | 70.92 |
| *Centropyge multifasciatus* | 0.51 | 0.78 | 0.39 | 3.72 | 74.64 |
| *60 m* |  |  |  |  |  |
| Average similarity: 15.58 |  |  |  |  |  |
|  |  |  |  |  |  |
| Species | Av.Abund | Av.Sim | Sim/SD | Contrib% | Cum.% |
| *Chromis ternatensis* | 1.42 | 2.38 | 0.41 | 15.29 | 15.29 |
| *Chromis alpha* | 0.99 | 2.36 | 0.63 | 15.14 | 30.43 |
| *Cephalopholis spiloparaea* | 0.62 | 1.29 | 0.40 | 8.28 | 38.71 |
| *Centropyge multicolor* | 0.51 | 0.94 | 0.42 | 6.02 | 44.73 |
| *Chromis acares* | 0.70 | 0.93 | 0.22 | 5.94 | 50.67 |
| *Labroides dimidiatus* | 0.48 | 0.90 | 0.41 | 5.78 | 56.45 |
| *Naso unicornis* | 0.65 | 0.89 | 0.29 | 5.68 | 62.13 |
| *Myripristes kuntee* | 0.58 | 0.83 | 0.30 | 5.30 | 67.44 |
| *Amblyglyphidodon aureus* | 0.45 | 0.62 | 0.31 | 3.99 | 71.43 |
| *Ctenochaetus striatus* | 0.47 | 0.46 | 0.21 | 2.95 | 77.65 |

b)

| *10 m* |  |  |  |  |  |
| --- | --- | --- | --- | --- | --- |
| Average similarity: 60.84 |  |  |  |  |  |
|  |  |  |  |  |  |
| Families | Av.Abund | Av.Sim | Sim/SD | Contrib% | Cum.% |
| Acanthuridae | 4.01 | 21.25 | 3.64 | 34.93 | 34.93 |
| Pomacentridae | 3.23 | 13.58 | 1.87 | 22.32 | 57.25 |
| Labridae | 2.44 | 9.89 | 1.70 | 16.26 | 73.51 |
| Chaetodontidae | 1.54 | 6.72 | 1.80 | 11.05 | 84.56 |
| *20 m* |  |  |  |  |  |
| Average similarity: 53.79 |  |  |  |  |  |
|  |  |  |  |  |  |
| Families | Av.Abund | Av.Sim | Sim/SD | Contrib% | Cum.% |
| Acanthuridae | 3.36 | 21.91 | 2.34 | 40.72 | 40.72 |
| Pomacentridae | 2.64 | 12.79 | 1.68 | 23.78 | 64.5 |
| Labridae | 1.73 | 9.70 | 1.81 | 18.03 | 82.53 |
| *30 m* |  |  |  |  |  |
| Average similarity: 53.74 |  |  |  |  |  |
|  |  |  |  |  |  |
| Families | Av.Abund | Av.Sim | Sim/SD | Contrib% | Cum.% |
| Pomacentridae | 3.41 | 20.98 | 3.32 | 39.05 | 39.05 |
| Acanthuridae | 2.71 | 13.09 | 1.62 | 24.37 | 63.42 |
| Labridae | 2.22 | 10.24 | 1.61 | 19.06 | 82.47 |
| *40 m* |  |  |  |  |  |
| Average similarity: 46.31 |  |  |  |  |  |
|  |  |  |  |  |  |
| Families | Av.Abund | Av.Sim | Sim/SD | Contrib% | Cum.% |
| Pomacentridae | 4.00 | 23.6 | 1.83 | 50.96 | 50.96 |
| Acanthuridae | 1.63 | 8.12 | 1.22 | 17.53 | 68.49 |
| Serranidae | 1.68 | 4.76 | 0.57 | 10.28 | 78.77 |
| *50 m* |  |  |  |  |  |
| Average similarity: 50.95 |  |  |  |  |  |
|  |  |  |  |  |  |
| Families | Av.Abund | Av.Sim | Sim/SD | Contrib% | Cum.% |
| Pomacentridae | 4.30 | 22.42 | 3.26 | 44.00 | 44 |
| Pomacanthidae | 1.35 | 6.41 | 1.85 | 12.59 | 56.59 |
| Labridae | 1.31 | 5.56 | 1.26 | 10.92 | 67.51 |
| Serranidae | 2.03 | 5.25 | 1.22 | 10.29 | 77.8 |
| *60 m* |  |  |  |  |  |
| Average similarity: 45.62 |  |  |  |  |  |
|  |  |  |  |  |  |
| Families | Av.Abund | Av.Sim | Sim/SD | Contrib% | Cum.% |
| Pomacentridae | 3.05 | 17.86 | 2.32 | 39.15 | 39.15 |
| Acanthuridae | 1.67 | 7.25 | 0.98 | 15.90 | 55.05 |
| Labridae | 1.37 | 6.27 | 1.21 | 13.75 | 68.8 |
| Pomacanthidae | 0.94 | 4.29 | 1.04 | 9.39 | 78.19 |

c)

| *10 m* |  |  |  |  |  |
| --- | --- | --- | --- | --- | --- |
| Average similarity: 74.57 | |  |  |  |  |
|  |  |  |  |  |  |
| Diet | Av.Abund | Av.Sim | Sim/SD | Contrib% | Cum.% |
| Herbivore | 4.94 | 29.09 | 4.60 | 39.01 | 39.01 |
| Planktivore | 3.62 | 19.94 | 3.31 | 26.74 | 65.75 |
| Mobile Invert Feeder | 2.39 | 11.71 | 1.92 | 15.71 | 81.45 |
| *20 m* |  |  |  |  |  |
| Average similarity: 67.74 | |  |  |  |  |
|  |  |  |  |  |  |
| Diet | Av.Abund | Av.Sim | Sim/SD | Contrib% | Cum.% |
| Herbivore | 3.99 | 30.67 | 2.37 | 45.28 | 45.28 |
| Planktivore | 2.77 | 17.63 | 1.81 | 26.02 | 71.3 |
| Mobile Invert Feeder | 1.69 | 11.26 | 2.02 | 16.63 | 87.93 |
| *30 m* |  |  |  |  |  |
| Average similarity: 63.70 | |  |  |  |  |
|  |  |  |  |  |  |
| Diet | Av.Abund | Av.Sim | Sim/SD | Contrib% | Cum.% |
| Herbivore | 3.32 | 23.92 | 2.99 | 37.55 | 37.55 |
| Planktivore | 3.81 | 23.82 | 1.92 | 37.40 | 74.95 |
| Mobile Invert Feeder | 1.90 | 9.10 | 1.26 | 14.29 | 89.24 |
| *40 m* |  |  |  |  |  |
| Average similarity: 61.77 | |  |  |  |  |
|  |  |  |  |  |  |
| Diet | Av.Abund | Av.Sim | Sim/SD | Contrib% | Cum.% |
| Planktivore | 4.75 | 31.96 | 2.59 | 51.74 | 51.74 |
| Herbivore | 2.19 | 13.64 | 1.68 | 22.08 | 73.82 |
| Mobile Invert Feeder | 1.26 | 7.31 | 0.99 | 11.84 | 85.66 |
| *50 m* |  |  |  |  |  |
| Average similarity: 67.75 | |  |  |  |  |
|  |  |  |  |  |  |
| Diet | Av.Abund | Av.Sim | Sim/SD | Contrib% | Cum.% |
| Planktivore | 5.68 | 32.42 | 3.77 | 47.84 | 47.84 |
| Herbivore | 2.17 | 13.39 | 2.11 | 19.76 | 67.6 |
| Mobile Invert Feeder | 1.83 | 10.61 | 1.77 | 15.66 | 83.27 |
| *60 m* |  |  |  |  |  |
| Average similarity: 67.18 | |  |  |  |  |
|  |  |  |  |  |  |
| Diet | Av.Abund | Av.Sim | Sim/SD | Contrib% | Cum.% |
| Planktivore | 4.50 | 37.25 | 3.07 | 55.45 | 55.45 |
| Mobile Invert Feeder | 2.46 | 19.15 | 1.83 | 28.51 | 83.96 |
