# Supplementary material for: Shifting reef fish assemblages along a depth gradient in Pohnpei, Micronesia
Source: PeerJ. 2018 Apr 24;6:e4650. doi: 10.7717/peerj.4650 (PMC5922234; doi:10.7717/peerj.4650)
Supplement: Table S6 — Summary statistics of the generalized linear mixed-effects models according to each depth contour. Note changes in the number of taxonomic groups reflects removal of ‘depth-generalists’ prior to analysis. [file peerj-06-4650-s006.docx]

**Table S6.** Summary statistics of the generalized linear mixed-effects models according to each depth contour. Note changes in the number of taxonomic groups reflects removal of ‘depth-generalists’ prior to analysis.

| Depth Contour | Family | Genus | Species | df | Log-likelihood | AIC | % Deviance explained |
| --- | --- | --- | --- | --- | --- | --- | --- |
| 30 m threshold | 44 | 108 | 223 | 6 | -125.0 | 261.9 | 9.6 |
| 40 m threshold | 43 | 106 | 223 | 6 | -113.4 | 238.8 | 12.0 |
| 50 m threshold | 46 | 110 | 239 | 6 | -109.0 | 230.0 | 8.5 |
| 60 m threshold | 47 | 123 | 264 | 6 | -103.4 | 218.8 | 8.2 |

df, degrees of freedom; AIC, Akaike information criterion
