# Supplementary figures and images for: Shifting reef fish assemblages along a depth gradient in Pohnpei, Micronesia
Source: PeerJ. 2018 Apr 24;6:e4650. doi: 10.7717/peerj.4650 (PMC5922234; doi:10.7717/peerj.4650)

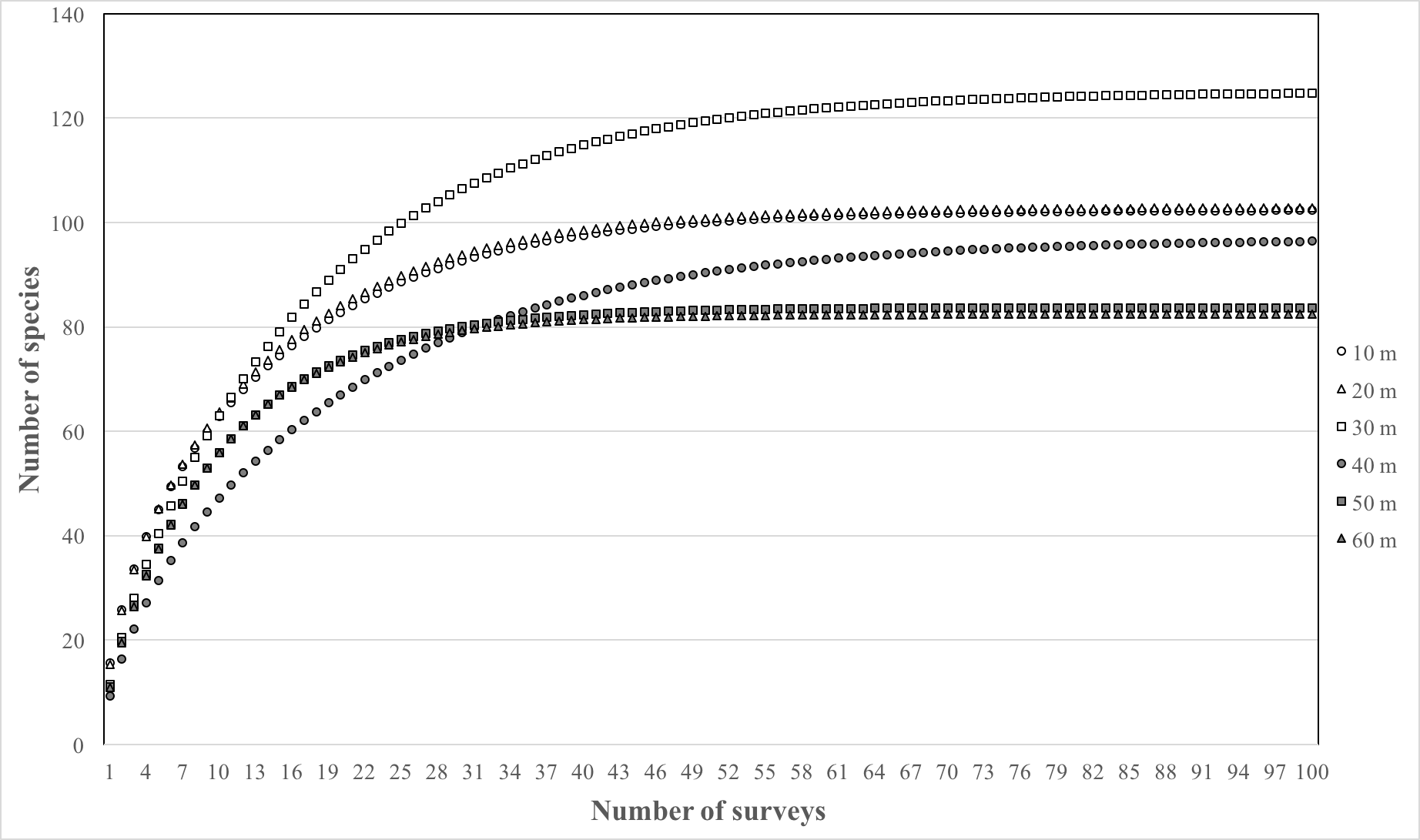

Supplement: Figure S1 — Species accumulation curve from fish surveys conduced between 10–60 m on the island of Pohnpei, Federated States of Micronesia. See inset for depth key. [file peerj-06-4650-s007.png]
